# Exploring induced pluripotency in human fibroblasts via construction, validation, and application of a gene regulatory network

**DOI:** 10.1371/journal.pone.0220742

**Published:** 2019-08-02

**Authors:** Mehdi B. Hamaneh, Yi-Kuo Yu

**Affiliations:** National Center for Biotechnology Information, National Library of Medicine, National Institutes of Health, Bethesda, Maryland, United States of America; University of Bonn, Bonn-Aachen International Center for IT, GERMANY

## Abstract

Reprogramming of somatic cells to induced pluripotent stem cells, by overexpressing certain factors referred to as the reprogramming factors, can revolutionize regenerative medicine. To provide a coherent description of induced pluripotency from the gene regulation perspective, we use 35 microarray datasets to construct a reprogramming gene regulatory network. Comprising 276 nodes and 4471 links, the resulting network is, to the best of our knowledge, the largest gene regulatory network constructed for human fibroblast reprogramming and it is the only one built using a large number of experimental datasets. To build the network, a model that relates the expression profiles of the initial (fibroblast) and final (induced pluripotent stem cell) states is proposed and the model parameters (link strengths) are fitted using the experimental data. Twenty nine additional experimental datasets are collectively used to test the model/network, and good agreement between experimental and predicted gene expression profiles is found. We show that the model in conjunction with the constructed network can make useful predictions. For example, we demonstrate that our approach can incorporate the effect of reprogramming factor stoichiometry and that its predictions are consistent with the experimentally observed trends in reprogramming efficiency when the stoichiometric ratios vary. Using our model/network, we also suggest new (not used in training of the model) candidate sets of reprogramming factors, many of which have already been experimentally verified. These results suggest our model/network can potentially be used in devising new recipes for induced pluripotency with higher efficiencies. Additionally, we classify the links of the network into three classes of different importance, prioritizing them for experimental verification. We show that many of the links in the top ranked class are experimentally known to be important in reprogramming. Finally, comparing with other methods, we show that using our model is advantageous.

## Introduction

Induced pluripotency (IP) in somatic cells, first achieved in mouse by Takahashi and Yamanaka [1], and then in human by Takahashi *et al*. [2] and Yu *et al*. [3], was a huge step forward for regenerative medicine. These studies demonstrated that somatic cells can be reprogrammed to induced pluripotent stem cells (iPSCs) by forced overexpression of certain transcription factors (TFs), referred to as reprogramming factors (RFs). OCT4 (also known as POU5F1), SOX2, KLF4, and MYC (collectively referred to as OSKM) constitute the most widely used set of RFs in IP experiments. But other factors, including NANOG and LIN28 (also known as LIN28A), have also been utilized in conjunction with some or all of the OSKM factors to generate iPSCs. Among these factors, OCT4, NANOG, and SOX2 are particularly important, because they form the core [4] of the pluripotency gene regulatory network (GRN). The RFs are thought to reprogram somatic cells by activating pluripotency-associated genes and by repressing somatic ones [5]. However, the underlying mechanism remains elusive despite the vast amount of accumulated research.

In many studies, a GRN has been constructed and used in conjunction with a model describing the network dynamics to provide insights into IP. However, most of these have employed a small network of known (curated) regulatory relations. Mitra *et al*. [6] described the dynamics of a single auto-regulating node by an ordinary differential equation (ODE). They showed that in the presence of an external input, the system can be driven from one steady state, i.e. somatic cell, to another, i.e. iPSC. Despite its simplicity, this study demonstrates the general idea behind many GRN-based models, i.e. reprogramming as a driven transition between two steady states. Garcio *et al*. [7] considered the three essential genes *OCT4*, *SOX2*, and *NANOG* and assumed 8 epigenetic states for each gene. The time evolution of the concentration of each possible gene state was then modeled using ODEs, assuming mass-action kinetics. The authors showed that their model is bistable and that the system can be driven from one state to another by adding exogenous SOX2 and OCT4. Using GRNs with four or five nodes (governed by ODEs) and adding epigenetic variables to the model, Miyamoto *et al*. [8] showed that pluripotency can be achieved from a differentiated state by overexpressing the pluripotency genes present in the model. Chickarmane and Peterson [9] used a GRN consisting of seven nodes and ODEs to provide a framework for exploring new strategies for IP. Employing ODEs, MacArthur *et al*. [10] proposed a model based on a GRN of 8 nodes, including the three core pluripotency genes and differentiation-specific genes. They demonstrated that, under certain circumstances, stochastic fluctuations in transcriptional status could result in reprogramming. Zhang and Wolynes [11] modeled cell differentiation by developing an analogy with quantum many-body problems. They wrote a master equation for the probability of the states of genes and, by applying the model to a GRN with 9 nodes, showed that the steady states of their model correspond to pluripotent and differentiated cells. Chang *et al*. [12] constructed a 52-node GRN by searching the literature for pluripotency-related regulations and used a dynamic Bayesian network, in which each node can be either on or off, for modeling gene regulations. This model produced results in good agreement with observed gene expressions after knockdown of OCT4 and suggested new recipes for pluripotency.

Unlike aforementioned studies that have used curated (and usually small) GRNs, Zhana *et al*. [13] built a much larger, 1625-node mouse GRN by fitting their proposed model (based on the work of Wang *et al*. [14]) to a time course microarray dataset obtained from a single mouse IP experiment. Assuming that TFs/genes with similar expression time courses are good candidates for having regulatory relations, the authors built a starting GRN and minimized a cost function to find the directions, signs, and weights of the links. They described the dynamics of the system using *linear* ODEs, and approximated the derivatives by differences.

The approaches taken by Chang *et al*. [12] and Zhana *et al*. [13] have useful features that are complementary. The former is capable of suggesting new RF combinations but uses a rather small curated network. The latter, specifically designed to infer a GRN, finds a large network, but it cannot suggest new IP recipes. In fact there are no parameters corresponding to the RFs in this model. On the other hand, in the method of [12] the variables corresponding to the RFs can take only two values (0 or 1), and so the model is not applicable to experiments in which the RFs are overexpressed at different levels. It is therefore desirable, albeit challenging, to have a GRN/model that goes beyond small curated networks, is capable of suggesting new RF combinations, and is able to account for RF stoichiometry. We circumvent these difficulties by building a rather large GRN (276 nodes) that provides all these desirable features.

To construct the GRN, we begin with a set of *non-linear* ODEs and find a relation between the (log-transformed) gene expression profiles of somatic cells and those of iPSCs. In this relation, the key role is played by a matrix whose elements encode the regulatory relations. We determine the signs (upregulation vs. downregulation) and the magnitudes (link strengths) of these matrix elements by fitting to a large set of experimental data. We test our GRN with an additional set of experimental data and find good agreement.

The constructed GRN may contain false positives, indirect links, or links that are not important for IP. To address this issue, we use a heuristic approach to classify the links based on their importance. We show that our GRN/model, unlike that of Chang *et al*. [12], can take into account the stoichiometry of the RFs and that our predictions regarding the effects of stoichiometry agree well with experimental observations. Additionally, we use the constructed GRN to suggest new RF cocktails for IP. Finally, since our approach is somewhat similar to that of Zhana *et al*. [13], we compare our GRN/model to theirs and show that using our GRN/model is advantageous. Note that since the majority of human iPSCs have been derived from fibroblasts, we limit our analysis to this cell type.

## Model

IP starts with forced overexpression of a few RFs that drive the cell out of its initial steady state (somatic cell) to the pluripotent state, after which the exogenous RFs are silenced [15] by *de novo* DNA methylation within iPSCs [16] and/or removed (completely) by other means [17]. We assume that the pluripotent state before the silencing/removal of exogenous RFs is another steady state allowed by the underlying dynamics.

At time *t* the state of the GRN, comprising *N* nodes, is characterized by the vector **E**(*t*) whose components are the expression levels of the genes. (We do not distinguish between a gene and its corresponding protein). The time evolution of **E**(*t*) is assumed to be described by the following set of *N* nonlinear ODEs [18]

$$\begin{array}{c} \frac{dE_{l}\left(t\right)}{dt}=R_{l}\left(E\left(t\right)\right)-\beta_{l}E_{l}\left(t\right),\;\;l=1,\;2,\ldots ,\;N. \end{array}$$ (1)

where *E*_*l*_(*t*) is the *l*th element of **E**(*t*), *R*_*l*_(**E**(**t**)) is a non-linear rate function and *β*_*l*_ is a constant. The first and second terms in Eq 1 represent gene regulation and protein degradation respectively. The function *R*_*l*_(**E**(*t*)) can be written as [19]

$$\begin{array}{c} R_{l}\left(E\left(t\right)\right)=\prod_{j=1}^{N}f_{lj}\left(E_{j}\left(t\right)\right), \end{array}$$ (2)

where *f*_*lj*_(*E*_*j*_(*t*)) is a regulation function describing how node *j* regulates node *l* (if no regulatory relation exists between the two then *f*_*lj*_(*E*_*j*_(*t*)) = 1). In the widely used Michaelis-Menten kinetics, for example, $f_{lj}(E_{j}(t))=f_{lj}^{max}(a_{lj}K_{lj}+b_{lj}E_{j}(t))/(K_{lj}+E_{j}(t))$ [19], where *K*_*lj*_ is a constant, $f_{lj}^{max}$ is the maximum rate, (*a*_*lj*_, *b*_*lj*_) = (0, 1) for upregulation, (*a*_*lj*_, *b*_*lj*_) = (1, 0) for downregulation, and (*a*_*lj*_, *b*_*lj*_) = (1, 1) with $f_{lj}^{max}=1$ for no regulation.

A somatic cell is stable and remains in this steady state until exogenous RFs are delivered. We introduce such a perturbation by multiplying the production rate of the node *l* by the constant *A*_*l*_, where *A*_*l*_ > 1 if node *l* is an RF and *A*_*l*_ = 1 otherwise. In other words, when the perturbation is turned on, the rate function changes to $R_{l}^{pert}(\text{E}(t))=A_{l}R_{l}(\text{E}(t))$.

Fitting the parameters in Eq 1 demands a large number of time course datasets generated using many different RF combinations. Unfortunately, one simply does not have this many time course datasets. For example, none of the 64 (Tables A and B in S1 File) experimental datasets used in this study contain such data. However, an important observation is that iPSCs share similar gene expression profiles regardless of which RF combination is used. This implies that the final expression profiles are universal and leads us to focus on steady states.

Noting that in any steady state the expression levels are time-independent, we use Eq 1 to write

$$\begin{array}{c} \frac{E_{l}^{\left(p\right)}}{E_{l}^{\left(i\right)}}=\frac{R_{l}^{\text{pert}}\left(E^{\left(p\right)}\right)}{R_{l}\left(E^{\left(i\right)}\right)}=A_{l}\frac{R_{l}\left(E^{\left(p\right)}\right)}{R_{l}\left(E^{\left(i\right)}\right)}, \end{array}$$ (3)

where the superscripts ^(*i*)^ and ^(*p*)^ denote respectively the initial somatic steady state and the pluripotent state before silencing/removal of exogenous RFs. Next we define the matrix **P** as follows

$$\begin{array}{c} P_{lj}\equiv \frac{{\text{log}}_{2}\left(f_{lj}\left(E_{j}^{\left(p\right)}\right)\right)-{\text{log}}_{2}\left(f_{lj}\left(E_{j}^{\left(i\right)}\right)\right)}{{\text{log}}_{2}\left(E_{j}^{\left(p\right)}\right)-{\text{log}}_{2}\left(E_{j}^{\left(i\right)}\right)}. \end{array}$$ (4)

Note that *P*_*lj*_ is positive (negative) if node *j* upregulates (downregulates) node *l* and vanishes otherwise.

Taking the logarithm of the two sides of Eq 3, we get

$$\begin{array}{c} W^{\left(p\right)}=PW^{\left(p\right)}+W_{s}, \end{array}$$ (5)

where the source vector **W**_*s*_ and the **W**^(*p*)^ vector have elements respectively given by $W_{s_{l}}={\text{log}}_{2}(A_{l})$ and $W_{l}^{\left(p\right)}={\text{log}}_{2}(E_{l}^{\left(p\right)}/E_{l}^{\left(i\right)})$. Assuming that **I** − **P** (**I** is the identity matrix) is invertible, Eq 5 can be written as

$$\begin{array}{c} W^{\left(p\right)}={\left(I-P\right)}^{-1}W_{s}. \end{array}$$ (6)

Before we can use Eq 6 we need to modify it slightly, because LIN28 upregulates *OCT4* post-transcriptionally [20]. In other words, upregulation of *OCT4* by LIN28 must be taken into account when computing the changes in expression levels of OCT4 targets, but it should be ignored as far as the expression level of *OCT4* is concerned. Eq 5 implies that the direct contribution of LIN28 to $W_{OCT4}^{\left(p\right)}$ is $P_{OL}W_{LIN28}^{\left(p\right)}$, where *P*_OL_ is the element of **P** that represents the regulation of *OCT4* by LIN28. Thus, we should replace $W_{OCT4}^{\left(p\right)}$ by $W_{OCT4}^{\left(p\right)}-P_{OL}W_{LIN28}^{\left(p\right)}$. Let $\tilde{\text{P}}$ be a matrix with elements ${\tilde{P}}_{lj}=P_{lj}$ for all *l* and *j* except when these indices correspond to OCT4 and LIN28 respectively, in which case ${\tilde{P}}_{lj}=0$. This implies that we should replace **W**^(*p*)^ by $W^{\left(p\right)}-(P-\tilde{P})W^{\left(p\right)}=(\tilde{P}+I-P)W^{\left(p\right)}=\tilde{P}{\left(I-P\right)}^{-1}W_{s}+W_{s}$.

The silencing/removal of the exogenous RFs, which is done after pluripotency is established [15–17], can be simply modeled by subtracting the source term **W**_*s*_ from the modified **W**^(*p*)^. Thus, we arrive at

$$\begin{array}{c} W=\tilde{P}{\left(I-P\right)}^{-1}W_{s}, \end{array}$$ (7)

where **W** is the vector of log-transformed fold changes between the final iPSC and the initial somatic state.

Despite its appearance, Eq 7 is *not* a *linear* relation as **P** depends on the initial and final gene expressions and varies by experiment. However, since iPSCs derived in different experiments have similar expression profiles (as do their parental fibroblasts), an effective yet somewhat universal, time-independent **P** (GRN) that takes the system from the start to finish can be envisioned even though IP is a dynamic process and regulatory relations may change during this process. Therefore, we look for an “average” **P** that is constant (the same for all experiments) and that can give results as close as possible to experimental data. In other words, we treat elements of **P** as constant parameters to be fitted. Note that even with this approximation the relation between the expression profiles of the fibroblasts and iPSCs is still non-linear as **W** contains the log-transformed fold changes.

Finally, one can calculate the predicted final expressions using

$$\begin{array}{c} {\text{log}}_{2}E={\text{log}}_{2}E^{\left(i\right)}+W, \end{array}$$ (8)

where log_2_ **E** is a vector whose elements are log_2_ *E*_*l*_.

We now turn to the source vector **W**_*s*_. Within an IP experiment, we write *A*_*l*_ = 2^*c*^ *S*_*l*_ for an RF represented by the node *l* in the network. While *S*_*l*_s are the *known* stoichiometric ratios of the RFs used, the *unknown* constant *c* is determined by fitting. As an illustration, consider an experiment where the level of OCT4 is three times those of the other RFs, we have *S*_OCT4_ = 3 for OCT4 and *S* = 1 for the others. In other words, we write

$$\begin{array}{c} W_{s}=cW_{s_{I}}+W_{s_{II}}, \end{array}$$ (9)

where the nonzero components of ${\text{W}}_{s_{I}}$ are all ones, and the *l*th component of ${\text{W}}_{s_{II}}$ is log_2_(*S*_*l*_). It should be noted that the only nonzero elements of **W**_*s*_, ${\text{W}}_{s_{I}}$, and ${\text{W}}_{s_{II}}$ are the ones that correspond to the RFs.

To summarize this section we note that if **P**, the stoichiometric ratios {*S*_*l*_}, and *c* are known, Eqs 7, 8 and 9 allow us to calculate the iPSC expression profile resulting from the reprogramming of fibroblasts whose expression profile is given by **E**^(*i*)^. Conversely, if ℳ experimental datasets (with known stoichiometric ratios) are available, one can use these equations to fit the elements of **P** and the set of coefficients {*c*_*m*_} (m=1,2…ℳ) provided that ℳ is large enough. In the following section we construct a human fibroblast reprogramming GRN by fitting these equations to a large number of experimental datasets. For details regarding the fitting process we refer the reader to the “Fitting and cross validation” section of Methods.

## Results and discussion

### Construction of the GRN

We constructed our reprogramming GRN by computing the matrix **P** via fitting Eq 7 to a large set of experimental data. However, to avoid prohibitively expensive calculations, only the nearest neighbors of the master regulators of pluripotency, i.e. OCT4/SOX2/NANOG, were considered, forming a reduced network of 276 nodes and 4471 links (“Constructing the starting network” section of Methods). To find **P**, we used this network in conjunction with 35 experimental gene expression datasets obtained from 20 Gene Expression Omnibus (GEO) [21] Series (“Experimental data” section of Methods and Table A in S1 File). Fitting was performed by minimizing the distance between the predicted (Eq 7) and experimental log-transformed fold changes. A repeated (10 times) 10-fold cross validation (100 fittings) was performed and the elements of **P** were computed by averaging the 100 fitted networks obtained from the 100 minimizations (“Fitting and cross validation” section of Methods). The nonzero elements of **P** and their uncertainties (the standard deviations computed from the 100 minimizations) are given in S2 File.

It is worth noting that only 7 out of the 35 experimental datasets used for fitting are independent (there are 6 distinct RF combinations and 1 OSKM study, from GEO Series GSE23583, in which OCT4 has been overexpressed more than the other RFs; see Tables A and B in S1 File). Hence, using the 35 datasets, the number of independent equations implied by Eq 7 is 1932 (7 × 276), which is less than half of the number of variables (number of links, 4471, plus the number of coefficients {*c*_*m*_}) present in the model. In other words, this is an underdetermined system. However, there are many constraints applied to the system, under which we find a unique solution. For details about these constraints and the rationale behind them see the “Fitting and cross validation” section of Methods. It is also worth mentioning that, although Eq 7 expresses **W** as a product of two matrices **W** = **QW**_*s*_, it is *not* just a matrix factorization. Unlike **P**, $Q=\tilde{\text{P}}(\text{I}-\text{P}{)}^{-1}$ is not sparse, and without a proper model relating **Q** to **P** the relation **W** = **QW**_*s*_ is useless in determining **P**, which is needed for construction of the GRN.

To the best of our knowledge our network is the largest constructed GRN for reprogramming of human fibroblasts and our study is the only one that has used a large set of experimental data to built such a GRN. There are, however, limitations for our model and the resulting GRN. Reprogramming is a dynamic process, but our model only relates the beginning (fibroblasts) and the end (iPSCs) of the process, resulting in an *effective* GRN that is expected to contain links that may be important only at some stages of reprogramming. Also, due to missing genes in the network, some of the regulatory links in the constructed GRN are likely to be indirect and some may not even exist (false positives). Despite these limitations, in the following sections we show that our model/GRN is capable of predicting log-transformed fold changes during the IP process as well as the experimentally observed trends of IP efficiency upon varying the stoichiometric ratios of the RFs. Additionally, we demonstrate that the model/GRN can be used in predicting new (i.e. not included in training of our model) RF combinations for IP, many of which have already been experimentally validated. We also address the issue of false positives in the “Link classes and subnetworks” section where we classify the links into three classes of different importance.

### Testing the model/GRN

In this subsection we test our model/GRN by: (1) comparing the predicted and experimental fold changes using 29 additional experimental datasets, and (2) investigating whether the effect of varying RF stoichiometry on our results is consistent with experimental observations.

#### Comparing predicted and experimental fold changes

We used 29 experimental datasets from 12 additional GEO Series as the “testing set” (see the “Experimental data” section of Methods and Table B in S1 File) to calculate the average correlation between the predicted (see “Computation of the predicted expression profiles” section of Methods) and experimental log-transformed fold changes. We found an average correlation of *r*^test^ = 0.8805, indicating good agreement between predicted and experimental values. In comparison, for the 35 training datasets the average correlation was *r*^train^ = 0.9292, which is (as expected) slightly higher. We also used goodness of fit, defined by *G* = 1 − *F*^a^, as another measure of comparison between the experimental and theoretical values. Here *F*^a^ is the cost function (see Eq 11) calculated using the average (indicated by superscript ^a^) fitted network. *G* was found to be 0.8712 and 0.7820 for the training and testing sets respectively, suggesting good agreement with experimental data. The closeness of *r*^train^ and *r*^test^ (as well as *G*^train^ and *G*^test^) suggests the absence of overfitting.

We also compared the predicted and experimental fold changes for an experiment performed using the RF cocktail OSK+PRDM14 (OSKP) [22], which was not used in the training of our model. We found the goodness of fit and the correlation between the theoretical and experimental values to be *G*^OSKP^ = 0.6119 and *r*^OSKP^ = 0.7751 respectively. These numbers are lower than the average values reported here for the testing set (*r*^test^ and *G*^test^), but they are within 3 standard deviations from these averages. In fact, *G*^*OSKP*^ is larger than $G_{min}^{test}=0.5740$ that is the minimum goodness of fit calculated for the 29 testing datasets. The correlation *r*^OSKP^ is also close to $r_{min}^{test}=0.8176$. Overall these results indicate reasonably good agreement between experimental and theoretical values for this RF cocktail, which was not used in the training of the model.

#### The effect of RF stoichiometry

In IP experiments with OSKM the efficiency of reprogramming has been reported to be dependent on the stoichiometric ratios of these RFs [23–26]. Here we show that the results of our model/GRN are largely consistent with these reports.

Since there is no theoretical measure that can be used to determine, for certain, whether a given expression profile is from an iPSC, we infer the reprogramming efficiency via a *probability* that we define below. Let **E**^th^, **E**^hESC^, and **E**^iPSC^ be vectors whose components are the predicted (by our model, Eq 8), human embryonic stem cell (hESC), and iPSC gene expression levels respectively. Experimental results suggest that the expression profiles of iPSCs are very close to those of hESCs. Therefore, if the distribution of *g* ≡ 1 − ((‖log_2_(**E**^iPSC^) − log_2_(**E**^hESC^)‖)/(‖log_2_(**E**^hESC^)‖))^2^ is known, one can assign a probability of representing an iPSC to any predicted log-transformed expression profile log_2_(**E**^th^) (‖•‖ denotes the L_2_ norm of •). Such a probability is simply calculated as the percentage of iPSCs for which g≤G, where $G=1-((\|{\text{log}}_{2}(E^{th})-{\text{log}}_{2}(E^{hESC})\|)/(\|{\text{log}}_{2}(E^{hESC})\|){)}^{2}$.

Using the available expression profiles of experimentally verified iPSCs and hESCs, the cumulative distribution of *g* can be approximated by computing a large number (denoted by *M*) of *g*s and sorting them in ascending order: *g*_1_ ≤ *g*_2_⋯≤*g*_*M*_. The probability for log_2_(**E**^th^) to represent an iPSC is defined as $q(G)=(1/M)[i+(G-g_{i})/(g_{i+1}-g_{i})]$ if $g_{i}\leq G<g_{i+1}$. Note that q(G)=0 and q(G)=1 for $G<g_{1}$ and $G\geq g_{M}$ respectively. For this study, *M* = 447 *g*s were calculated using the iPSC and hESC samples included in the 32 GEO Series listed in Tables A and B in S1 File. Note that not all included GEO Series contain hESC samples. To avoid errors that may arise when comparing data from different Series, only iPSC and hESC samples published in the same Series were used for these calculations (Series not containing any hESC sample were excluded in the calculation of the probability *q*).

To measure how RF stoichiometry affects IP efficiency, we use the equal stoichiometry (O:S:K:M = 1:1:1:1) point as the reference. Fifty-eight fibroblast samples (Tables A and B in S1 File) are used as the initial somatic states for calculating the 58 corresponding log_2_(**E**^th^) profiles (“Computation of the predicted expression profiles” section of Methods). With the aforementioned procedure, we calculated the 58 corresponding probabilities (“Computation of probability *q*” section of Methods) and denote their average by ${\bar{q}}^{Ref}$, our reference probability of the 1:1:1:1 stoichiometry for producing an iPSC.

**Fig 1:**
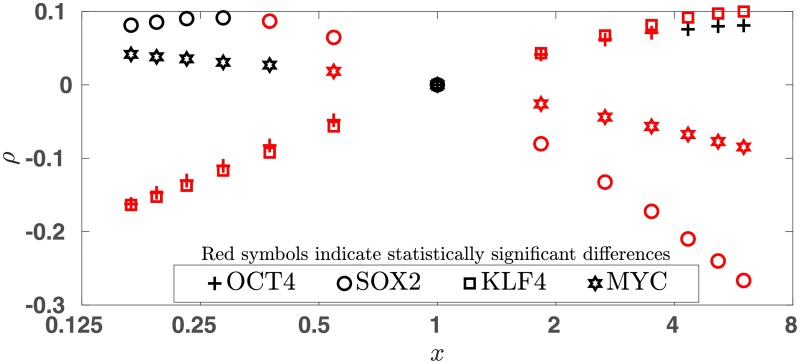
Effect of stoichiometry. For different stoichiometric ratios (O:S:K:M) *ρ*(*x*), defined in Eq 10, are plotted. Here *x* is the relative decrease or increase in the level of one of the RFs while those of the rest of the RFs remain unchanged. A positive (negative) *ρ* is suggestive of a higher/lower IP efficiency.

Using this approach one can calculate a set of 58 probabilities *q*_*j*_ (*j* = 1, 2…, 58) and their average $\bar{q}$ for any given stoichiometry (or in general for any RF cocktail). To assess the effect of stoichiometry on $\bar{q}$, we varied one at a time the levels of the RFs by a factor of 1/6 ≤ *x* ≤ 6 and calculated $\bar{q}(x)$ for each RF. For the 4 RFs we then calculated *ρ*(*x*) defined as

$$\begin{array}{c} \rho \left(x\right)\equiv \left(\bar{q}\left(x\right)-{\bar{q}}^{\text{Ref}}\right)/{\bar{q}}^{\text{Ref}}, \end{array}$$ (10)

which are plotted in Fig 1. A positive (negative) *ρ* indicates a larger (smaller) $\bar{q}$ compared to the reference, suggesting efficiency increase (decrease). To see if the difference $\left({\bar{q}}^{F}(x)-{\bar{q}}^{Ref}\right)$ is statistically significant, in each case we performed a t-test, see Methods section. Whenever the difference is significant at the 0.01 level, the corresponding point is shown in red. As detailed below, our results show overall good qualitative agreement with the experimentally observed efficiency trends:

*Increased/decreased level of OCT4* (the + symbols): Our model predicts a lower efficiency when OCT4 is expressed less than the other RFs and an increased efficiency with increased levels of OCT4, although the increased efficiency is statistically significant only up to a point. This agrees with what Papapetrou *et al*. [23] observed.
*Increased/decreased level of SOX2* (the circles): We find reduced efficiency for higher SOX2 levels in agreement with Papapetrou *et al*. [23] and increased efficiency when the level of SOX2 is lowered, albeit the increase is statistically significant only when *x* is close to unity. Experimentally, Papapetrou *et al*. [23] and Yamaguchi *et al*. [24] both reported an improvement in efficiency with lowered SOX2 level, but [23] found the efficiency increase to be small and statistically insignificant. Examining our model prediction at *x* much smaller than 1 and at *x* close to but smaller than 1, provides a possible explanation for the different observations by [23] and [24] respectively.
*Increased/decreased level of KLF4* (the squares): We find a lower efficiency with lower KLF4 levels in agreement with [23, 25], although [23] reported a small (and statistically insignificant) decrease. The reported experimental efficiency trends due to increased levels of KLF4 are conflicting. Although Papapetrou *et al*. [23] found a significantly lower efficiency, Kim *et. al*. [26] reported an improved efficiency in mouse reprogramming experiments. Our result agrees with that of [26].
*Increased/decreased level of MYC* (the hexagrams): In agreement with our findings, Papapetrou *et al*. [23] reported a significant efficiency decrease when the level of MYC was increased relative to the other RFs. On the other hand, [23] observed no significant efficiency change with MYC level lowered, which is what we predict for most values of *x* < 1 (for one value of *x* shown in the figure we find a small but significant efficiency increase).

### Link classes and subnetworks

As mentioned previously, our GRN may contain false positive links and therefore false variables. One way to limit the number of false variables is to penalize the number of variables introduced. Such a scheme results in networks whose numbers of links depend on the magnitude of the penalty term. We opted not to add such a term to our cost function because minimizing a function containing a discrete term is computationally too expensive, if not impossible, in the current context. Instead we limited the number of variables based on available evidence (“Constructing the starting network” of Methods) and devised a method to construct smaller subnetworks that presumably contain fewer false positives but have comparable performance to the whole network (hereafter referred to as WN).

To construct such subnetworks, we first assign (see the “Link scores” section of Methods) a score *s*_*lj*_ to the link from node *j* to node *l*. The link score is defined as the change in the cost function upon removal of that link. The link ranking, inevitably, depends on the fitted network: changes in the number or strengths of the links are likely to change the ranking. We nonetheless show that by removing links with scores less than a cutoff and re-fitting the remaining links one can construct subnetworks that produce results in good agreement with experimental observations. The cutoff, however, cannot be too large as it results in a subnetwork too sparse to give good results. For example, using the cutoff value 10^−2^ yields a subnetwork of 105 links with the goodness of fit being 0.4172 (0.3897) for the training (testing) set. This is significantly lower than the value found for WN (0.8712/0.7820 for the training/testing set). Therefore, we chose two smaller cutoffs *δ*_1_ = 10^−3^ and *δ*_2_ = 10^−4^ to construct two subnetworks, referred to as SUBN1 and SUBN2 respectively, that have significantly lower numbers of links than WN but give results that are comparable to that of WN. The link strengths for these two subnetworks (given in S2 File) were found via the same procedure used for fitting WN. The number of links *L*, goodness of fit *G*, and average correlation *r* for these subnetworks and WN are given in Table 1. It is clear from the table that links with *s* < 10^−4^ collectively contribute little to the cost function and removing all of them has almost no effect on the predicted gene expression. Therefore, we did not use cutoffs smaller than *δ*_2_ = 10^−4^.

**Table 1:** Performance comparison between WN and the subnetworks.

Network	*L*	*G*^train^	*G*^test^	*r*^train^	*r*^test^
SUBN1	445	0.8263	0.7646	0.9036	0.8700
SUBN2	1078	0.8650	0.7792	0.9257	0.8788
WN	4471	0.8712	0.7820	0.9292	0.8805

*L*, *G*, and *r* denote number of links, goodness of fit, and average correlation respectively.

**Fig 2:**
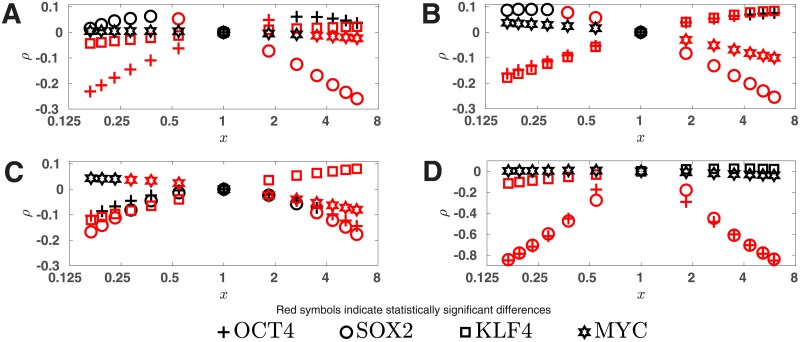
Effect of stoichiometry if the subnetworks are used. For different stoichiometric ratios (O:S:K:M) *ρ*, defined in Eq 10, are plotted as a function of *x* for (A) SUBN1, (B) SUBN2, (C) *C*_1_ ∪ *C*_3_, and (D) *C*_2_ ∪ *C*_3_. Here *x* is the relative decrease or increase in the level of one of the RFs while those of the rest of the RFs remain unchanged. *C*_*k*_ denotes the set of links in Class *k* (see text for definition). SUBN1 and SUBN2 are the two subnetworks comprising links in *C*_1_ and *C*_1_ ∪ *C*_2_, respectively. A positive (negative) *ρ* is suggestive of a higher/lower IP efficiency.

Table 1 also indicates that the two subnetworks can predict the log-transformed fold changes with good accuracy (measured by *G* and *r*). Although the accuracy improves as the number of links increases, the improvement is small when comparing SUBN1 with SUBN2 and even smaller when comparing SUBN2 with WN. To further investigate the performance of SUBN1 and SUBN2, we next examined whether they can predict the experimentally observed IP efficiency trends. Using the procedure described in the previous section, we calculated *ρ* for different stoichiometric ratios and the results are plotted in Fig 2A and 2B for SUBN1 and SUBN2 respectively. Comparing these figures with Fig 1 it is clear that SUBN2 and WN predictions are quite close and that SUBN1 also shows qualitatively the same trends although for some *x* values the networks do not agree on the statistical significance of *ρ*. Since SUBN1 and SUBN2 have comparable performance to that of WN but contain significantly fewer links, they are expected to have fewer false positives than WN.

The results above lead us to classify the links into 3 classes denoted by *C*_*k*_ (*k* = 1, 2, 3): *C*_*k*_ contains links whose scores satisfy *δ*_*k*_ < *s*_*lj*_ ≤ *δ*_*k*−1_, with *δ*_0_ = max({*s*_*lj*_}), *δ*_1_ = 10^−3^, *δ*_2_ = 10^−4^, and *δ*_3_ = min({*s*_*lj*_}). The 3 classes *C*_1_, *C*_2_, and *C*_3_, respectively have 445, 633, and 3393 links (class assignments are given in S2 File). Note that SUBN1 consists of links in *C*_1_, SUBN2 comprises links in *C*_1_ ∪ *C*_2_, and WN contains all links (*C*_1_ ∪ *C*_2_ ∪ *C*_3_). The results summarized in Table 1 suggest that in SUBN2 members of *C*_2_ are less likely than those in *C*_1_ to be important in the IP process. Similarly, these results indicate that in WN, *C*_*k*+1_ is collectively less important than *C*_*k*_ (because adding *C*_*k*+1_ to the network results in a smaller accuracy improvement than adding *C*_*k*_). However, it should be emphasized that this importance assignment is conditional, i.e. *C*_*k*_ is more important than *C*_*k*+1_ *if* *C*_*k*_ is present in the GRN. To investigate what would happen if the GRN contained *C*_*k*+1_ but not *C*_*k*_, we constructed the other 4 possible subnetworks that may be built using the 3 link classes, i.e. subnetworks consisting of links in *C*_2_, *C*_3_, *C*_1_ ∪ *C*_3_, and *C*_2_ ∪ *C*_3_. The link strengths of these 4 subnetworks were computed by the same fitting and cross validation procedure as used for WN and SUBN1/SUBN2. We found that some of these 4 subnetworks give *G*s and *r*s comparable to those of WN/SUBN1/SUBN2 (Table C in S1 File). However, as shown in Fig 2C and 2D (for *C*_1_ ∪ *C*_3_, *C*_2_ ∪ *C*_3_) and also in Fig A in S1 File (for *C*_2_, *C*_3_), we observed that none of the 4 subnetworks can predict the IP efficiency trends as well as WN, SUBN1, or SUBN2 (compare these figures with Fig 1 or Fig 2A and 2B). These findings indicate that only subnetworks built by adding the classes in a certain order can produce results that agree well with the experimentally observed efficiency trends. Based on these findings and those summarized in Table 1 we conclude that links in *C*_*k*_ are more likely to be important than those in *C*_*k*+1_ for reprogramming and that our classification is appropriate.

**Fig 3:**
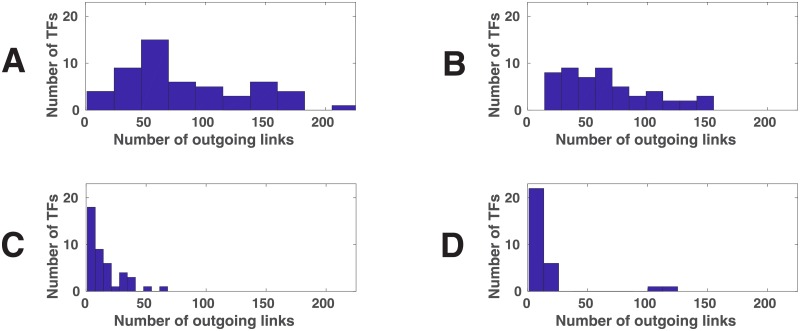
Link distribution in different classes. The distribution of the number of outgoing links per node is shown for (A) WN, (B) *C*_3_, (C) *C*_2_, and (D) *C*_1_, where *C*_*k*_ denotes the set of links in Class *k* (see the text for the definition of the link classes). The figure indicates that the distributions of links in *C*_2_ (panel C) and *C*_1_ (panel D) are much more non-uniform in comparison with that of links in *C*_3_. In *C*_1_ and *C*_2_ OCT4 and SOX2 have the largest number of outgoing links. The gap between these two TFs and others is especially large in *C*_1_ (panel D), indicating the importance of OCT4 and SOX2 in *C*_1_.

We also looked at the distribution of the number of links per node in these 3 classes as well as the whole network. The results, shown in Fig 3, indicate that the distribution of the links in the whole network (Fig 3A) and in *C*_3_ (Fig 3B) are much more uniform than in *C*_2_ (Fig 3C) and *C*_1_ (Fig 3D). Specifically, in *C*_1_ (Fig 3D) there are two nodes that have more than 100 outgoing links and the rest of the nodes have 21 or less. Not surprisingly, these two nodes are OCT4 and SOX2. This is what we expect to observe in the most important Class as OCT4 and SOX2, in addition to being in the core of pluripotency circuitry, are the most widely used RFs and so their links must be the most important ones. Interestingly, in *C*_1_ NANOG (with 18 links) is ranked 5th after KLF4 (19 links) and ZIC3 (21 links). (Note that *ZIC3* is also a pluripotency gene [27]). This suggests that NANOG may not be as important as OCT4 and SOX2, although it is, along with OCT4 and SOX2, a part of the core regulatory network of pluripotency in hESCs. This finding may explain why NANOG has been used much less frequently than OCT4, SOX2 for reprogramming. As another RF used in the training of our model, MYC, with 10 links, is ranked 12th in *C*_1_. It is worth noting that, in our GRN, LIN28 has only 1 outgoing link to OCT4. We could not find other experimentally verified or even inferred links for LIN28. This is a limitation of our GRN (but not of our model).

In *C*_2_ (Fig 3C), OCT4 is again separate from the rest of the pack (with 68 links), but the separation is not as big as it is in *C*_1_. In fact, KLF4 (50 links), NANOG (41 links), SOX2 (40 links), FOXO1 (38 links) and MYC (31 links) are not far behind. In *C*_3_ (Fig 3B), on the other hand, none of the 6 RFs used in the training of our model is among the top 10. The highest ranking one is NANOG with 91 links (ranked 14th). OCT4 (35 links) and SOX2 (33 links) are ranked 41st and 43rd respectively. Overall the results shown in Fig 3 indicates that OCT4 and SOX2 become more important (have higher relative number of links) in comparison to other nodes in *C*_2_ and especially in *C*_1_, suggesting again that *C*_*k*_ is more likely than *C*_*k*+1_ to contain important reprogramming links.

As a test for our link classification method, we also investigated the effect of randomly shuffling the genes (the rows of the matrix **W**) on the performance of our model. If all genes (nodes) in the starting network are connected to each other, the nodes will be equivalent and shuffling the data should not significantly change the performance of the model. In other words, the two performance measures (average correlation and goodness of fit) should not change significantly if one uses randomized, rather than real, data. On the other hand, if the use of prior biological knowledge in constructing the starting network is successful in reducing the number of false positives, we should observe a lower performance in the case of random data. Additionally, if our classification method is successful, the difference in performances (when random vs. real data are used) should be even more pronounced in the subnetworks (because they will have fewer false positive links). As detailed below, this is exactly what we observed.

We did the shuffling 100 times for the training set and re-fit the link strengths, i.e. we performed minimizations and cross validations 100 times and constructed 100 networks. For each of these 100 networks we then compared the predicted fold changes with the randomly generated data that was used to construct the network. In other words, for each of the 100 networks the two performance measures *r* (correlation) and *G* (goodness of fit) were computed. We performed this whole procedure for WN as well as SUBN1 and SUBN2. In all three cases, the 100 networks constructed using the randomized data performed worse than the ones built using the real data. In the case of WN the two measures *r* and *G*, respectively, ranged from 0.8678 to 0.9276, and from 0.7675 to 0.8685. Table 1 indicates that even the maxima of these values (0.9276 and 0.8685) are smaller than the ones obtained using the real data (*r*^train^ = 0.9292 and *G*^train^ = 0.8712), although the differences are not large. As expected, we observed much larger performance differences using the subnetworks. Specifically, we found 0.7957 ≤ *r* ≤ 0.8998 and 0.6543 ≤ *G* ≤ 0.8203 when randomized data were used in conjunction with SUBN2. We observed even lower values, i.e. 0.5858 ≤ *r* ≤ 0.7649 and 0.3700 ≤ *G* ≤ 0.6058, for SUBN1. Comparing these values with the ones obtained using the real data given in Table 1, we find that the decreases in correlation and goodness of fit (due to randomization) is largest when SUBN1 is used. The effect is also larger in the case of SUBN2 as compared to WN. These findings suggest that our classification method is successful and that *C*_*k*_ is more likely than *C*_*k*+1_ to contain important links.

There are a few points worth mentioning regarding our link classification: (1) As previously mentioned, the individual link scores are network-dependent. For example, the rank correlation (measured by Kendall’s Tau) between the two score sets calculated using WN and SUBN1 for links in *C*_1_ is low (0.37). However, we have shown that *C*_1_ is more important than the other classes for producing acceptable results both in presence and in absence of the other classes. In other words, removing *C*_3_ and *C*_2_ does not change the fact that Class 1 is more important, but it significantly alters the ranking of its individual links. Link classes are also network-dependent, but to a much less extent. For example, defining new classes $C_{1}^{SUBN2}$ and $C_{2}^{SUBN2}$ using SUBN2 (instead of WN) to compute the link scores, we found that 77% (79%) of *C*_1_ (*C*_2_) links were are also in $C_{1}^{SUBN2}$ ($C_{2}^{SUBN2}$), indicating a good overlap. Overall these results suggest that the link scores are helpful for classifying links in classes, but they are not good enough for ranking the links individually. (2) Although we explained the rationale behind our choice of cutoffs, the number and magnitude of the cutoffs, and thus the number of classes and their membership, are to a large extent arbitrary. However, our goal is not to develop a rigorous classification algorithm but to offer a pragmatic classification for prioritizing links for experimental verification and for finding smaller subnetworks (that are likely to have fewer false positives) with comparable performance to that of WN. (3) Ranking *C*_*k*_ higher than *C*_*k*+1_ does not mean that every link in the former is more important than all links in the latter. Instead this ranking suggests that overall links in *C*_*k*_ are more likely to be important than those in *C*_*k*+1_.

#### Biological validation of links in SUBN1

We have shown that *C*_1_ links, which constitute SUBN1, are collectively more important than the rest of the links for predicting experimental observations. We have also shown a network without *C*_1_ links cannot predict the correct IP efficiency trends. Therefore, it is useful to take a closer look at SUBN1 and to investigate whether the *C*_1_ links are supported by prior biological knowledge. We first note that 137 out of 445 links in SUBN1 are known direct targets/regulators of OCT4, SOX2, NANOG, or PRDM14 in hESCs and thus in iPSCs (for references see S2 File). Since these 4 TFs are essential in maintenance of pluripotency [4, 22, 28], their direct links in hESCs are likely to be important in reprogramming. As indicated in the “Constructing the starting network” section of Methods, we refer to these as Group 1 links, denoted by *T*_1_, and the rest of the links are assigned to Group 2, denoted by *T*_2_ (see S2 File for the lists of links in *T*_1_, *T*_2_, and *C*_1_ ∩ *T*_1_). Note that this categorization of links has nothing to do with the classification proposed here and that not all links in *T*_1_ are members of *C*_1_. However, the percentage of Class 1 links that are also in Group 1 is 31%, which is significantly higher than those of Class 2 (18%) and Class 3 (4%), indicating *C*_*k*_ is more likely than *C*_*k*+1_ to include biologically important links.

**Fig 4:**
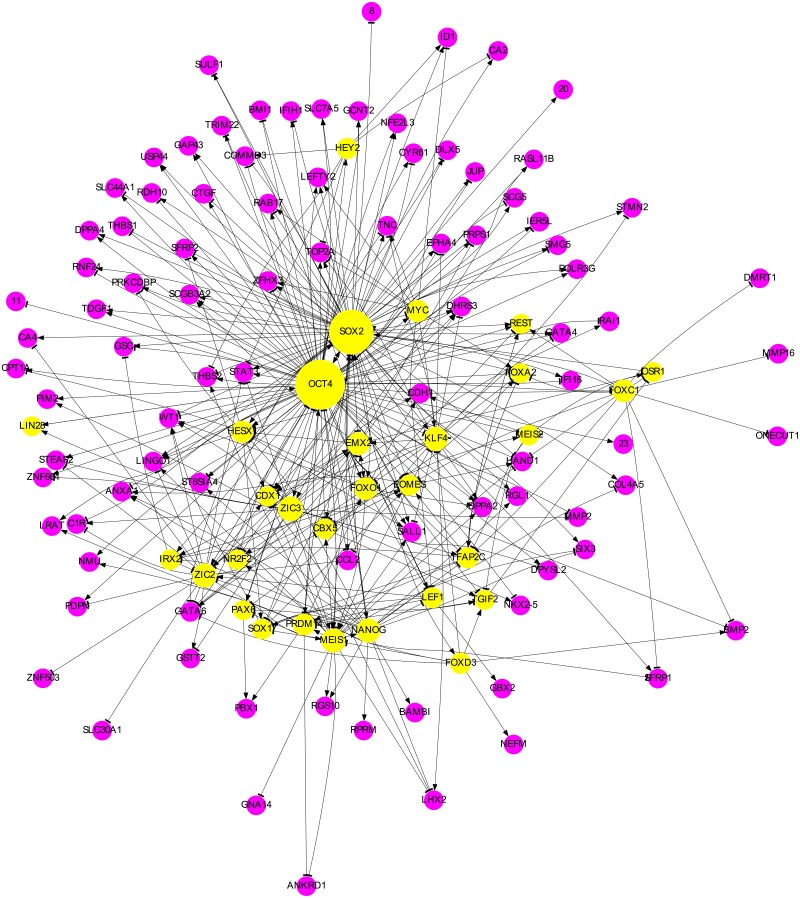
The SUBN1 network. The regulating nodes are shown as yellow circles whose radii increase linearly with the number of outgoing links. In magenta are the nodes that, in this subnetwork, do not regulate other nodes. Each magenta node that has only a numeric label *k* represents a collection of *k* nodes regulated in the same way. A “tee” arrowhead denotes downregulation, while a “normal” one indicates upregulation. If two nodes mutually regulate each other, only one link is shown with an arrowhead at each end.

The largest connected component of SUBN1, shown in Fig 4, has 179 nodes, making the network effectively smaller (some nodes are disconnected in this subnetwork). Thus, we investigated if these nodes are of importance in the IP process. We found that more than half (99 out of 179) of these genes undergo a 4 fold change or more when averaged over the 64 experimental datasets (Tables A and B in S1 File). In comparison, among the 97 disconnected nodes only 5 have fold changes larger than 4. Many of the 99 genes with large fold changes are well-known pluripotency genes [27] such as *DPPA4*, *LEFTY2*, *ZIC3*, and *TDGF1* that need to be upregulated. Others may be involved in a pathway that is important for pluripotency. For example, expression of *DKK1* has been shown to inhibit Wnt signaling that is important for IP [29], and so its downregulation by OCT4 plays an important role. On the other hand, in some cases like upregulation (about 80 fold when averaged over the 64 datasets) of *NMU* and downregulation (about 8 fold on average) of *SULF1*, we have not yet found literature evidence on the role of these genes in IP. Interestingly, the only Group 1 link to *SULF1* is a positive one from OCT4 (not included in Class 1). Note that the signs of Group 1 links are known from the literature (see “Constructing the starting network”) and were fixed during the fitting process (“Fitting and cross validation” section of the Methods). The fact that *SULF1* is highly downregulated requires at least a negative Group 2 link to *SULF1*. Indeed a link from SOX2 exists in SUBN1 that not only counters the effect of OCT4 but also downregulates *SULF1*. This example shows at least some of Group 2 links are important for producing the right expression profiles. In fact, when we constructed a network consisting of only the 137 links in *C*_1_ ∩ *T*_1_ and fitted them using our procedure, we found a much lower goodness of fit (*G* = 0.5011) compared to that of SUBN1 (Table 1). This demonstrates the importance of including the Group 2 links when constructing the starting network.

Let us note that we could not confirm (or reject) any of the *C*_1_ ∩ *T*_2_ links via literature search. This is partly because we did an extensive literature search while constructing the starting network and included whatever we found in *T*_1_. Lacking a real gold standard to validate *C*_1_ ∩ *T*_2_ links, we chose an indirect validation method. We treated some of the links in *C*_1_ ∩ *T*_1_ as if they were in *T*_2_ (i.e. we did not fix their signs) and re-fitted WN. We then investigated whether our classification method still assigns these links to *C*_1_. Specifically, the *C*_1_ ∩ *T*_1_ links were randomly partitioned into 10 subsets. They were then moved, one subset at a time, to Group 2 (while the other 9 subsets remained in Group 1), the corresponding **P** matrices were found by fitting, and in each case the links were classified using our method. We performed this whole procedure 10 times, resulting in 100 (10 × 10) GRNs and thus 100 new classifications. We found that, on average, 78% of the links moved to Group 2 were assigned to the new Class1 after re-fitting. Also, 78% of these links were assigned correct signs by the fitting procedure. These results suggest that our fitting process yields good sign assignment and good link classifications. However, the best way to verify *C*_1_ ∩ *T*_2_ links (see S2 File), be they direct or effective, is by performing experiments. In fact suggesting links for experimental verification is a main goal of the current study.

### Suggested new RF combinations

Encouraged by the overall good agreement between our results and experimental observations, we used our model for suggesting “new” (i.e. not used in training of our model) RF combinations for IP. We first compiled a list of RF candidates. An RF must be able to provide significant feedback to the core of the GRN. Thus, we considered a list comprising of OCT4/SOX2/KLF4/MYC/NANOG (already confirmed RFs) and any TF in our GRN that directly regulates the core (OCT4/SOX2/NANOG) via a link in Class 1. The identified candidate RFs, in addition to the already confirmed RFs, were EMX2, FOXC1, HESX1, LEF1, MEIS1, PRDM14, ZIC2, and ZIC3. We did not include LIN28 in the list as it has only one outgoing link in our network (post-transcriptional regulation of OCT4). Hence, overexpression of LIN28 results in that of OCT4, meaning that in our GRN LIN28 can replace OCT4 in most RF combinations, which is not the case experimentally. This limitation of our GRN is due to the lack of evidence of other possible targets of LIN28. More experimental data are required to truly reveal the role of LIN28 in our GRN.

We used the members of the aforementioned list in groups of 1, 2, 3, or 4 as RFs (overexpressed at the same level) and calculated, using SUBN1/SUBN2/WN, the predicted log-transformed expression profiles for each combination as well as the 58 probabilities {*q*_*j*_} described earlier. We then ranked the RF cocktails based on the average probability $\bar{q}$. Out of 1092 RF combinations tested using WN, 136 had nonzero $\bar{q}$ (Table D in S1 File). Results from using SUBN1 and SUBN2 are also available (Tables E and F in S1 File). Excluding the RF combinations used in the training data, the three sets of top 10 suggested RF combinations, calculated using SUBN1, SUBN2, and WN respectively, are given in Table 2. The three sets of RF cocktails are *largely* similar, suggesting a good agreement among the 3 networks.

**Table 2:** Suggested RF combinations.

Rank	1	2	3	4	5	6	7	8	9	10
WN	OS	OSM	OSN	OSKN	OSNM	OSKP	OSP	OSNP	OSMP	OSKZ
SUBN2	OS	OSM	OSN	OSKP	OSNM	OSP	OSKN	OSMP	OSNP	SKM
SUBN1	OS	OSM	OSNM	OSN	OSKN	OSNP	OSKP	OSMH	OSKH	OSP

Abbreviations: O: OCT4, S: SOX2, K: KLF4, M: MYC, P: PRDM14, Z: ZIC3, and H: HESX1.

A literature search showed that 6 out of the top 10 (including 4 out of the top 5) RF cocktails suggested by WN i.e. OS [30], OSN (OS+NANOG) [3], OSKN [31], OSNM [32], OSK+PRDM14 (OSKP) [22], and OSMP [22] have been already experimentally verified. However, OSM has been reported to be unable to produces iPSCs in mouse [1]. Interestingly, OSK+ZIC3 (OSKZ) have been successfully used as RFs in IP experiments in mouse [33], but in human it has been reported to reprogram fibroblasts to neural progenitors [34]. Note that instead of OSKZ, SUBN2 suggests SKM, which like OSKZ is a false positive [1]. On the other hand, SUBN1 (when compared to WN) suggests OSM+HESX1 (OSMH) and OSK+HESX1 (OSKH) in place of OSKZ and OSMP. We could not find literature support for these two combinations (OSMH and OSKH), but OKM+HESX1 has been shown to produce iPSCs when combined with some other factors [35], confirming HESX1 as an RF. We failed to find experimental confirmation (or rejection) for other cocktails listed in Table 2. However, the unconfirmed RF sets are in fact more interesting, because they are natural candidates for experimental verification.

One should be careful when comparing the ranks of the suggested RF sets with the corresponding experimentally observed relative efficiencies. Specifically, efficiencies obtained using different experimental protocols cannot be compared to each other. This is because reprogramming efficiency does not solely depend on RFs used. Experimental details and the RF delivery method also affect the efficiency of IP [36, 37]. Therefore, to study how IP efficiency varies due only to change in the employed set of RFs, one needs to compare results obtained from the same experimental protocol. Keeping this in mind, we searched the literature for information regarding the relative efficiencies of the confirmed RF cocktails listed in the table. The results of our literature search can be summarized as: *e*(OSKM) > *e*(OSK) [38], *e*(OSKM) > *e*(OSNM) [32], *e*(OSK) > *e*(OS) [30], *e*(OSKP) > *e*(OSK) [22], and *e*(OSKN) ∼ *e*(OSK) [31], where *e*(XYZ) denotes the efficiency of IP if XYZ are used as RFs. (Note that using our model the OSKM and OSK RF combinations rank higher than other cocktails but they are not listed in the table because they are used in our training dataset. See Tables D, E, and F in S1 File). A comparison between these 5 experimental observations and our results indicates that our model correctly predicts the first 3.

Given the fact that our method ranks OSKM the highest in terms of predicted efficiency one might ask what the point is of suggesting other RF cocktails with lower efficiencies. Obviously, this ranking is predicted and, in some cases, may be wrong (For example, OSKP has been shown to reprogram with a higher efficiency than OSK). Much more importantly, efficiency is not the only concern when deciding what RF set to use. There are other reasons why one may want to choose other RF combinations. The fact that, despite high efficiency of OSKM, many experimental studies have used other RF sets demonstrates an interest in alternative approaches for practical purposes. For example, MYC is a well-known oncogene. Therefore, other factors have been used in its place to produce safer iPSCs (see, for instance, [38]). As another example, consider the study that showed OSKP is capable of reprogramming fibroblasts [22]. This study was performed primarily to shed light on the molecular mechanisms of the reprogramming process, which are still poorly understood today.

As mentioned previously, we used a selected list of TFs to suggest new RF combinations given in Table 2. This was necessary to remove a large number of false positives. To explain why absence of such selection criteria leads to numerous false positives, let us give an example. Consider the cocktail OSKX (OSK+X), where X is an arbitrary TF in the network. If X provides little or no feedback to the network, the gene expression, and so $\bar{q}$, predicted for OSK and OSKX will be almost identical. In other words, our model would rank OSK and OSKX at the same level and suggests OSKX as a new RF cocktail. Obviously, a TF that cannot affect the network may not produce iPSCs and thus any RF combination that includes X is a false positive. Note that the reverse is not true, i.e. if OSKX and OSK have the same $\bar{q}$, we cannot conclude that X does not affect the GRN or that OSKX is a false positive. For example, it has been experimentally shown that OSK and OSKN have comparable efficiencies [31]. It is thus not possible to find false positives by comparing $\bar{q}$ values. The only way to avoid these false positives is to restrict considered TFs to the ones that provide significant feedback to the network. Therefore, we applied the aforementioned selection criteria for RF candidates. Note that we excluded LIN28 for the same reason, i.e. to avoid false positives. In the case of LIN28, as mentioned previously, false positives arise from the fact that in our network LIN28 has only 1 outgoing link (to OCT4). This is a limitation of our GRN (but not of our model). However, this limitation only reflects the fact that we could not find more experimentally verified or even inferred links for LIN28.

Although, to reduce the number of false positives, it was necessary to restrict the list of potential RFs, such restrictions may bias the results towards previously known RFs. Therefore, we repeated our search for suggested RF combinations (as described above) without any restrictions on the RF candidates. We tested all possible combinations of (up to 4) RFs (a total of 317682 combinations) and used WN for the calculations. Out of these 317682, we found 16861 RF cocktails with $\bar{q}>0$, which are given in S3 File. Note that the six previously mentioned experimentally verified RF sets (OS, OSN, OSKN, OSNM, OSKP, and OSMP) are among top 1.5% in the complete list. We would like to emphasize again that the complete list given in S3 File contains a lot of false positives, but at the same time it may provide more new ideas as compared to the list given in Table 2.

### Which network to use

The results of the previous sections indicate that the three networks (WN, SUBN1, and SUBN2) give comparable performance in suggesting new RF sets, predicting the efficiency trends, and predicting the fold changes (although the goodness of fit for SUBN1 is a bit lower). In other words, it appears that for practical calculations one may use any of these networks. However, using a larger network has two effects: it is more likely to contain false positive links but less likely to have false negative links.

### Performance comparison

Although numerous methods have been proposed to infer GRNs from experimental data (for example, see [39] and references therein), to the best of our knowledge only one method, proposed by Zhana *et al*. [13], has been specifically proposed for constructing a large (> 100 nodes) GRN for reprogramming. Therefore, we only compare the performance of our method to that of Zhana’s. This method, based on the approach taken by [14], is somewhat similar to ours as it finds the GRN by minimizing a cost function. However, there are significant differences. Most importantly, Zhana’s method does not include any parameters corresponding to the RFs and their cost function is different from ours. Also, seeking a sparse solution, their cost function includes a penalty term (the sum of absolute link strengths multiplied by a positive parameter λ). The parameter λ controls the number of nonzero links, denoted by *L*, in their GRN: higher λ means lower *L*. We refer to a fitted network with *L* links by Zhana’s method as ZN*L* (for example, ZN1075 denotes a network with 1075 links).

Zhana’s method was developed to use time course expression data. Unfortunately, datasets that include expression profiles of intermediate states between human fibroblast and iPSC are very rare. However, all experimental datasets used in this study can be regarded as a time series consisting of two time points: the initial (fibroblast) and the final (iPSC) states. For this reason, and also to have a fair comparison, we used our 35 training datasets for fitting and the additional 29 datasets for testing Zhana’s method. The same starting network described in the “Constructing the starting network” section of Methods was used for fitting Zhana’s model.

**Fig 5:**
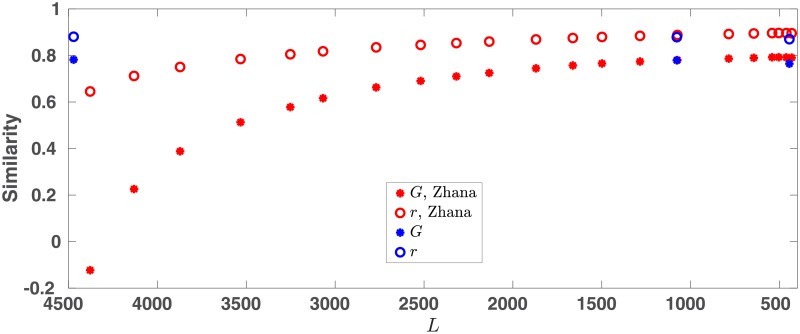
Comparing the two methods. The average correlation *r*, and goodness of fit *G* are plotted as functions of number of links using both our method (in blue) and Zhana’s (in red). Note that SUBN2 and ZN1075 have almost the same number of links (1078 and 1075 respectively) and give almost identical results, and so the blue symbols are on top of the red ones.

We performed the calculations for 0.01 ≤ λ ≤ 60, and used the aforementioned similarity measures (average correlation, *r*, and goodness of fit, *G*) to compare the predicted and experimental (from the 29 testing datasets) log-transformed fold changes. Since different λs correspond to different networks with varying number of links, in Fig 5 we plot the similarity measures as a function of *L*. For comparison, we also plot the results of our method obtained using WN, SUBN1, and SUBN2 (Table 1).

The figure indicates that, unlike our method that gives comparable results using all 3 networks, the performance of Zhana’s method depends on *L* and deteriorates as *L* increases (λ decreases). For small values of λ (*L* ≳ 3000), our method provides significantly better results. Interestingly, SUBN2 and ZN1075 (obtained with λ = 10) have almost the same number of links (1078 vs. 1075) and give very close *r* and *G* (the corresponding points in the figure are on top of each other).

For λ > 10, i.e. *L* < 1075, the methods are comparable, although Zhana’s method seems slightly better. However, a careful look revealed that ZN432 and SUBN1 (with *L* = 445) share only 50 links, indicating a significant difference between them. Upon further investigation we found that ZNL with *L* < 1075 appear to miss some important links based on available experimental evidence. For example, PRDM14 has been reported to have an important role in pluripotency and has been successfully used as an RF in reprogramming of human fibroblasts [22, 28]. However, in ZNL with *L* < 1075, PRDM14 has very few targets and they do not regulate any other nodes. In such networks PRDM14 is unlikely to induce pluripotency. In comparison, as shown in the previous section, calculations performed with SUBN1 suggests PRDM14 as a new RF (Table 2). As another example, ZIC2 is reported to have an important role in maintaining pluripotency [40], but it does not regulate any node in ZNL with *L* < 1075. On the other hand, in SUBN1, ZIC2 regulates 18 nodes and provides feedback to the core pluripotency genes. Therefore, ZNL with *L* < 1075 (λ > 10) seems to disagree with some experimental evidence. On the other hand, for λ ≤ 10, both similarity measures obtained using ZNL are either comparable to or worse than the ones given by our method.

Even if/when the two models are comparable (in terms of predicting the expression profiles) our method has an important advantage. The model developed by Zhana *et al*. can be used only for inferring gene regulatory relations, whereas our model, in addition to inferring the GRN links, can suggest new RF combinations and/or stoichiometric ratios for IP. To the best of our knowledge, the method proposed by Chang *et al*. [12] is the only one that can take RFs as parameters and has the ability to predict new RF cocktails, though their model cannot take into account the RF stoichiometry and uses a small curated GRN. Unfortunately, the code implementing the approach of [12] was unavailable, making it difficult to compare their method with ours. Nonetheless, even absent a performance comparison our method has the clear advantage of being able to take into account the RF stoichiometry.

## Methods

### Experimental data

From the Gene Expression Omnibus (GEO) database [21], we collected data, obtained using platform GPL570, from reprogramming of human fibroblasts. We chose GPL570 and fibroblasts because they are widely used. We looked for GEO Series containing raw microarray data of both the derived iPSCs and the parental fibroblasts. Experiments using RFs not present in our starting network (see “Constructing the starting network” section) were excluded with few exceptions. In some studies factors such as hTERT and SV40LT have been used in addition to the RFs to facilitate the process. However, these factors have been shown to have only an indirect and non-essential role in induction [41]. Thus, we included such studies even though the additional factors are not included in our network. We found 32 GEO Series satisfying our criteria. In each GEO Series the iPSC samples derived from the same type of fibroblast using the same experimental procedure were grouped together, and so were their corresponding fibroblast samples. Each iPSC group and its corresponding fibroblast group constitute a “dataset”. If an iPSC cell line was included (perhaps for comparison purpose) in multiple Series, we assigned it to only one dataset. Some GEO Series contain multiple experiments using different fibroblast types and/or experimental protocols, resulting in multiple datasets. Overall 64 datasets were included in our analysis, out of which 35 were used for training in a repeated cross validation (CV), as described in the “Fitting and cross validation” section, and the rest (29 datasets) were employed for testing. A detailed description of the included datasets and the corresponding references are given in Tables A and B in S1 File for the training and testing sets respectively. The raw data from each dataset were processed using the robust multi-array average (RMA) algorithm to find the expression levels of the probesets and their corresponding genes. If a gene had multiple corresponding probesets, their expression levels were averaged. In each dataset the samples in the iPSC (fibroblast) group were averaged resulting in a single expression profile for iPSC (fibroblast).

To split the 64 datasets into training and testing sets, we noted three points. First, to avoid overfitting, it is important to have as many different RF cocktails as possible in the training set. So, we made sure every RF cocktail used in these 64 datasets is represented in the training set. Second, datasets published in the same GEO Series may not be completely independent. Although such datasets differ in some way (for example, reprogramming with different sets of RFs), they share some other important experimental details, for example use the same parental fibroblasts, or employ the same experimental design, etc. Therefore, to make sure the testing and training sets are totally independent, we did not split a GEO Series. In other words, all datasets in a Series were assigned to either the testing or training set. Third, the vast majority of the RF cocktails used in these experiments contain KLF4 and MYC. Specifically, out of the 64 experiments, 59 (51) have used KLF4 (MYC). Thus, to avoid a potential bias against NANOG and LIN28, we decided to assign most (2/3) of the datasets using NANOG or LIN28 to the training set.

Based on all these observations, we split the 32 GEO Series as follows. We assigned all Series using OS+NANOG+LIN28 (OSNL) to the training set, because these are the only ones not using KLF4 or MYC (these include 5 datasets, see Table A in S1 File). We also included the 1 Series using OSKML in the training set. Similarly, the 3 Series containing 3 datasets using OSKMN, were assigned to the training set. The OSKMNL is the cocktail with the largest number of associated experiments (8 datasets included in 3 Series) among the ones using NANOG or LIN28. Therefore, datasets employing OSKMNL were split between the training and testing sets as follows: 2 datasets were included in the training set and the rest were used for testing. This procedure resulted in a 67/33% (training/testing) split of the 18 datasets that have used NANOG or LIN28. The rest of the Series were randomly divided between the training and testing sets in such a way that each set has 19 OSKM datasets and 4 OSK datasets (a 50/50% split). As mentioned previously, this whole procedure resulted in 35 and 29 datasets in the training and testing sets respectively (a 55/45% split; see Tables A and B in S1 File).

### Constructing the starting network

**Fig 6:**
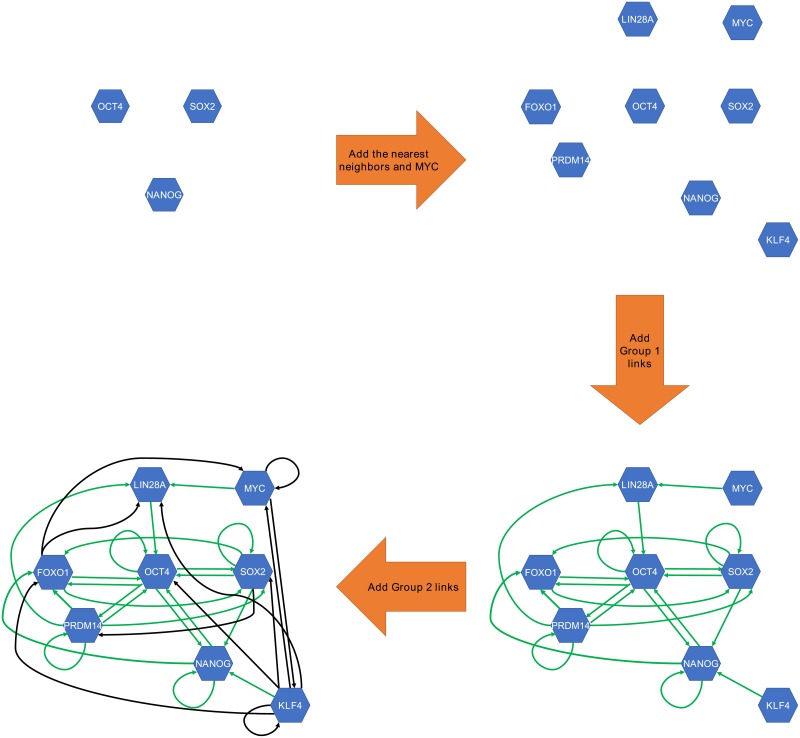
Construction of the starting network. Using a small representative subset of nodes, the figure explains how the starting network was constructed. We started from OCT4, SOX2, and NANOG and built the network around these TFs by adding their experimentally verified direct targets and regulators and MYC (see the text for details). We then connected the nodes of the network by adding regulatory links in two steps. First, the experimentally verified links (Group 1; colored in green) were added. These included the links between OCT4/SOX2/NANOG and their targets/regulators as well as other experimentally verified links that we found. We then added a large number of inferred links (Group 2; colored in black) based on a database developed by Marbach et al. [42].

As mentioned in the introduction, the previously constructed human reprogramming GRNs are small. This is because of the huge computational cost of fitting the model parameters when the GRN is large. Although our intention here is to construct a much bigger network compared to previous works, we still need to limit the size of the GRN in terms of the number of nodes and links. Obviously, when a selection like this is necessary, it must be done based on prior biological knowledge. A common way for obtaining the necessary prior knowledge is conducting a literature search (for example, many of the works cited in the introduction have used this approach). We perform this selection process in two main steps: (1) selecting the nodes to include in the GRN, and (2) adding links between the selected nodes. The second step in turn is performed in two stages: (1) adding experimentally verified links, and (2) including inferred links. Fig 6 summarizes this whole process. In the figure a small sample of nodes are shown to represent the network.

For the first step, i.e. deciding which nodes to include, we note the following experimental findings: (1) iPSCs are almost identical to hESCs, (2) OCT4, SOX2, and NANOG constitute the core of the regulatory network responsible for maintaining pluripotency in hESCs [4], and (3) these three are well-established RFs, although NANOG has been much less frequently used as an RF (Tables A and B in S1 File). Note that the second point gives OCT4, SOX2, and NANOG special status that no other TF has. Although there are other genes important for pluripotency, like PRDM14 [22, 28], none of these genes are considered part of the core. It could be argued that NANOG does not have the same status as OCT4 and SOX2, because it has been less frequently used as an RF and also because replacing NANOG (in OSNM) by KLF4 increases the efficiency of reprogramming [32]. Nonetheless, since NAONG has been shown to be a part of the core pluripotency circuitry, we decided to build the starting network around OCT4, SOX2, and NANOG. In other words, we included, as nodes, the direct targets and regulators of these factors in hESCs. But we also added the established RFs LIN28, KLF4, and MYC.

To identify the direct targets or regulators of the core (OCT4, SOX2, and NANOG) in hESCs, we conducted a literature search. During our literature search we found two types of study. Some papers, based on their experimental findings, specifically name a handful of genes as the targets or regulators of the core [43–49]. We included all these targets and regulators in the network. On the other hand, we found three large scale knockdown experiments of OCT4, SOX2, and/or NANOG. Note that up or downregulation of a gene after knocking down OCT4, for example, does not necessarily mean the gene is a direct target of OCT4. The change in the expression of the gene could just be an indirect effect. Boyer et al. [4] have published lists of potential targets of the core factors based on their binding sites. Thus, in the case of these three knockdown studies we considered a gene a direct target of OCT4, for example, if that gene was in the Boyer list of potential OCT4 targets and it had undergone a more than 1.5 fold change in at least one of the knockdown experiments. Out of the three knockdown studies two [50, 51] have gene expression data available, and so we computed the average fold changes from the expression data collected three or more days after the knockdown. For the other knockdown experiment [52], we used the reported fold changes (after three days). At this point we had completed adding the targets and regulators of the core. Since KLF4 and LIN28 were already in the network, we added MYC (see the previous paragraph). This resulted in a network of 276 nodes.

The next step was to connect the nodes. We first added the experimentally verified links that we found during our literature search. These included links connecting the core TFs to their direct targets or regulators. However, we found other links while performing the search, including regulation of LIN28 by MYC [53] in hESCs and several direct targets of PRDM14 [22, 54] in hESCs. One of the papers studying PRDM14 [22] reports a knockdown experiment with additional experimental data on PRDM14 biding sites. In this case, again we considered a gene a direct target of PRDM14 if PRDM14 had been found to bind to the gene and the gene had undergone at least 1.5 fold change upon knockdown of PRDM14.

In rare cases our literature search found conflicting results regarding the nature of the links (upregulation vs downregulation). Such links were removed. At this point our starting network had 400 links, which we refer to as the Group 1 links (represented by green links in the lower half of Fig 6). We distinguish these links from any other added to the network, because these are supported by strong experimental evidence and their signs (upregulation vs downregulation) are known.

A network consisting of only Group 1 links has many missing links, because it does not include links between most of the TFs present in the network and their targets. Unfortunately, experimentally verified regulatory relations are not available for the vast majority of the TFs. An alternative is to add all links to the network, i.e. connect each TF to all other nodes. Since 87 out of the 276 nodes are considered TFs [55], the number of all links is 20412. Obviously, many of these links are false positives, because TFs regulate only a fraction of genes, not all of them. On the other hand, fitting such a large number of links require a lot of computational power. Both of these scenarios, i.e. keeping only the 400 links in Group 1 or adding all links, are impractical extremes. Thus, we opted for something in the middle, i.e. adding all inferred (based on TF sequence motif) links. Therefore, we used a database, developed by Marbach et al. [42] (http://regulatorycircuits.org), of inferred type- and tissue-specific regulatory networks. Note that, due to regulatory changes during reprogramming, an average GRN that takes the system from a somatic state to a pluripotent one is likely to include links that are not present in hESCs, or even in fibroblasts. Therefore, we included all links suggested by this database regardless of tissue or cell type.

After including links from Marbach et al. [42], which are referred to as Group2 links (represented by black links in Fig 6), we reached a final list of 4471 links (S2 File). Note that Group2 links are not supported by knockdown experiments and are only inferred, without any specific relation to pluripotency. Hence these links are considered much less reliable in comparison to those from Group 1. Also note that by using this database we reduced the number of links 4.5 times (from 20412, i.e. the number of all possible links, to 4471). Our procedure thus removed a large number of false positives. However, many false positives may still exist in the network. This is exactly why we developed our heuristic algorithm for classifying the links (described in the “Link classes and subnetworks” section of Results) that allowed us to identify links that are more likely to be important in the IP process.

### Fitting and cross validation

Given a starting network and a set of training data, the following cost function was minimized

$$\begin{array}{c} F=\left\langle F_{m}\right\rangle =\langle {\left(\frac{∥W_{m}^{\text{th}}-W_{m}^{\text{exp}}∥}{∥W_{m}^{\text{exp}}∥}\right)}^{2}\rangle , \end{array}$$ (11)

where 〈•〉 is the average of • over the included datasets, ‖•‖ denotes the L_2_ norm of •, $W_{m}^{th}$ (given by Eq 7) is a vector containing predicted log-transformed fold changes corresponding to the *m*th dataset, and the *l*th component of $W_{m}^{exp}$ is defined as $W_{lm}^{exp}={\text{log}}_{2}E_{lm}^{iPSC}/E_{lm}^{FIB}$. Here *E*_*lm*_ represents the expression level of *l*th node from the *m*th dataset. Note that in addition to the nonzero elements of **P**, the coefficients *c*_*m*_ (defined in Eq 9) are also unknowns in our model and are determined by fitting. The L-BFGS-B algorithm [56] was used in conjunction with SciPy’s [57] “minimize” function which requires a specified tolerance (denoted by *ϵ*). Letting the cost function evaluated at step *k* be *F*(*k*), the routine stops when [*F*(*k*) − *F*(*k* + 1)]/max{|*F*(*k*)|, |*F*(*k* + 1)|, 1}≤*ϵ*. Each minimization was subject to the following constraints: (1) *P*_*lj*_ = 0 unless *j* was a candidate regulator of *l*, (2) the sign of each Group 1 link was fixed, (3) |*P*_*lj*_| < 1 (In a system governed by Michaelis-Menten kinetics one can show |*P*_*lj*_| < 1 for all *l* and *j*, which we assume throughout the analysis.), (4) *c*_*m*_ > 0. For more details about the minimizations and their convergence/robustness see the “Robustness and convergence” subsection below.

We performed a repeated (10 times) 10-fold cross validation (CV) for all minimizations. Every time the 35 training datasets were randomly divided into 10 batches each comprising 3 or 4 datasets. Each batch was then left out while the other 9 were grouped together and used in the minimization. Thus, for a given list of starting values, 100 minimizations were run resulting in 100 fitted networks, which were then averaged to find what we refer to as the average fitted network.

#### Computation of the predicted expression profiles

Once an average fitted network is found, for any given initial expression profile (fibroblast) and any given source term (**W**_*s*_), one can use Eqs 7 and 8 to calculate the predicted expression levels of the resulting iPSC. However, if only the stoichioemtric ratios are known, **W**_*s*_ remains unknown because it depends on the unknown variable *c* (Eq 9), which can vary from experiment to experiment. To address this issue, we note that the goal of an IP experiment is to derive cells that are as close as possible to hESCs. Given an hESC expression profile (or any reference profile) denoted by **E**^REF^, one can find an optimal *c* that minimizes ‖**W**^th^ − **W**^REF^‖, where $W_{l}^{REF}={\text{log}}_{2}(E_{l}^{REF}/E_{l}^{FIB})$. Such a *c* is given by

$$\begin{array}{c} c=\frac{\left(W_{I}^{\text{th}},W^{\text{REF}}-W_{II}^{\text{th}}\right)}{∥W_{I}^{\text{th}}{∥}^{2}}, \end{array}$$ (12)

where $W_{I}^{th}=\tilde{\text{P}}(\text{I}-\text{P}{)}^{-1}W_{S_{I}}$, $W_{II}^{th}=\tilde{\text{P}}(\text{I}-\text{P}{)}^{-1}W_{S_{II}}$, and with (*X*, *Y*) denoting the dot product of *X* and *Y*. Usually the reference profile is that of an hESC, but given an iPSC expression profile one can use Eq 12 (with **W**^REF^ = **W**^iPSC^) to find the *c* that produces the closest profile to that of the iPSC. In this paper, we have used Eq 12 to find *c* whenever it was needed to compute the predicted fold changes using an average fitted network. Note that if in an experiment the RFs are overexpressed at the same level, $W_{II}^{th}=0$ and thus **W**^th^ is proportional to $W_{I}^{th}$. Hence, in such a situation one can find the correlation between the predicted and the experimental log-transformed fold changes without knowledge of *c* (this is the case for all 29 datasets chosen for testing our method).

#### Robustness and convergence

We first ran a set of 100 minimizations using the starting network described in the “Constructing the starting network” section with *ϵ* = 10^−8^ and for a set of initial values. The initial *c* values for different datasets were chosen to be the same, denoted by *c*_0_. The initializing procedure for the link strengths is described later in this subsection. The resulting fitted *c*_*m*_ values were all close to the initial value *c*_0_, suggesting that the cost function is a slowly varying function of *c*_*m*_ and that one set of minimizations may not be enough to find the optimal network. Therefore, we repeated the minimizations (and cross validation) for various *c*_0_ values ranging from 2 to 18.

For a given *c*_0_ the average fitted network was found by averaging the link strengths over the 100 minimizations. Note that the fitted *c*_*m*_ values obtained from the set of 100 minimizations were not averaged. Instead, to find the *c*_*m*_ values corresponding to an average fitted network we used Eq 12 with $W_{m}^{REF}=W_{m}^{iPSC}$. The predicted log-transformed fold changes $W_{m}^{th}$ (*m* = 1…35) were computed and were subsequently fed to Eq 11 to find the cost function corresponding to the average network, denoted by *F*^a^(*c*_0_). Here the superscript ^a^ indicates that the cost function was computed using the average fitted network and the argument *c*_0_ shows that the average fitted network was found using the initial value *c*_0_.

We found that *F*^a^(*c*_0_) indeed varies slowly as a function of *c*_0_ with a shallow minimum at *c*_0_ = 12 (Table G in S1 File). On the other hand, further analysis showed (Table G in S1 File) that for *c*_0_ ≥ 8, the link strengths of the corresponding average fitted networks have very high correlations and rank correlations with the network obtained using *c*_0_ = 12. In other words, for *c*_0_ ≥ 8 the obtained fitted average networks are very similar (in terms of the relative strengths of the links). It is worth noting that since *A*_*l*_ = 2^*c*^ (if the RFs are overexpressed at the same level), values of *c* that are close to zero or are much higher than 10 are not likely to be used in practice. This is because a small *c* indicates only modest increases in the production rates of the RFs, whereas *c* = 18, for example, means hundreds of thousands fold increase in the production rates. Based on these observations we chose *c*_0_ = 12 for all minimizations used in this study.

In the minimizations the initial link strengths $P_{0_{lj}}$ were chosen using the following procedure. For the links in Group 2 the initial values were set to zero whereas for the Group 1 links they were chosen randomly. This choice was made because there are no information available on the signs of Group 2 links, and hence choosing random signs for these links may produce biased results. Additionally, many of these links may not even exist and so setting their initial values to zero appears to be reasonable.

For each node *j*, regulating at least one node, we also subjected the initial values $P_{0_{lj}}$ to the following constraint:

$$\begin{array}{c} \sum_{l}\left|P_{0_{lj}}\right|=d, \end{array}$$ (13)

where *d* is a random number between zero and unity. This constraint forces the initial link strengths to be less than 1, but it is more restrictive than $|P_{0_{lj}}|<1$ and guarantees that all eigenvalues of the matrix **P**_0_ have magnitudes smaller than 1. This constraint was applied because we found that in its absence the minimization algorithm has difficulty finding the minimum. We ran 10 sets of 100 minimizations with the constraint given by Eq 13 applied (with different initial values for Group 1 links), and another 10 sets requiring $|P_{0_{lj}}|<1$ instead. For each set we computed the average link strengths, and then calculated the correlations between different sets of average link strengths. We found that the 45 pairwise correlations between the 10 sets of link strengths obtained under the constraint defined by Eq 13 were all larger than 0.9602 (with an average of 0.9914), indicating that the corresponding 10 sets of minimizations have converged to practically the same point (in terms of relative strengths of the links). On other hand, we observed that when Eq 13 was not applied the results were dependent on the initial strengths of Group 1 links (the 45 pairwise correlations ranged between 0.4882 and 0.7485). The 100 pairwise correlations between average link strengths obtained in the presence and in the absence of the constraint (given by Eq 13) were also low, ranging from 0.2526 to 0.3635. More importantly, we found that the goodness of fit was always higher when Eq 13 was applied in comparison to minimizations without this constraint (Table H in S1 File). These results suggest the constraint represented by Eq 13 must be applied to find the minimum. Interestingly, we observed that in all 10 cases |λ_*m*_| < 1 (|λ_*m*_| > 1) if Eq 13 was (not) applied, where λ_*m*_ is the eigenvalue of the average fitted matrix **P** with the largest absolute value. This provides an explanation as to why applying Eq 13 as a constraint is useful (note that all minimizations for this purpose were performed with *c*_0_ = 12).

The convergence of the minimizations was also investigated by running minimizations with different tolerances ranging from 10^−2^ to 10^−8^. We observed a monotonic increase in correlation (Spearman rank correlation) between link strengths corresponding to *ϵ* = 10^−*i*^ and *ϵ* = 10^−(*i*+1)^ from 0.6399 (0.5591) to 0.9765 (0.9746) for *i* ≥ 3, indicating reasonably converged results.

Averaging over 100 minimizations also has the advantage that it provides a measure of uncertainty for each obtained link strength. We use the standard deviation of the 100 values for each link as its uncertainty.

### Computation of probability *q*

Suppose the expression profile **E**^FIB^ of the parental fibroblasts and the stoichiometric ratios of the RFs are given. One can use Eqs 7, 8, 9 and 12 to calculate the predicted expression profile log_2_(**E**^th^) that is closest to a given ${\text{log}}_{2}(E_{i}^{hESC})$. However, since the expression profiles of different hESC cell lines (or different replicates of the same cell line) are slightly different, the coefficient *c* and hence log_2_(**E**^th^) vary slightly depending on the reference hESC profile used. In other words, if there are *m* hESC samples in an included GEO Series, for a given **E**^FIB^ in this Series one finds *m* slightly different predicted expression vectors, each corresponding to an hESC profile. One can then compute $G_{i}=1-((\|{\text{log}}_{2}(E_{i}^{th})-{\text{log}}_{2}(E_{i}^{hESC})\|)/(\|{\text{log}}_{2}(E_{i}^{hESC})\|){)}^{2}$ (for *i* = 1, 2, …, *m*) and the corresponding probability $q(G_{i})$. For the fibroblast sample considered, the probability of turning into iPSCs (under the IP procedure) is assumed to be the average of these *m* probabilities.

### Link scores

A simple way to rank a link in a given network is to assess the impact of the removal of the link on the network. In a GRN such a removal is likely to result in a change in the expression profile and consequently in the cost function (see Eq 11). Presumably, a larger change indicates a higher degree of importance for the removed link. Suppose an averaged fitted network has been obtained and let *F*^a^ be the cost function corresponding to this network and calculated using the 35 training datasets. One may compute $F_{km}^{a}$ that is the cost function when the link *km* is removed from the network while the rest of it remains intact. The “score” $s_{km}=F_{km}^{a}-F^{a}$ can then be assigned to the link *km*. It is worth mentioning that by definition the magnitudes of the scores are all less than 1 and they are generally very small. This is because there are many links in the network, making the change due to removal of one link small. What is important in this analysis is the ranking of the scores and not their magnitudes.

### Statistical significance

For every point shown in Figs 1 and 2, and Fig A in S1 File, the statistical significance of *ρ*, defined in Eq 10, was determined using a two-sided, one-sample t-test. Specifically, in each case the null hypothesis was that *ρ* is from a normal distribution with a mean equal to zero. Given the large number of points in each sample (i.e. 58) the assumption of the normal distribution and thus the use of a t-test is reasonable.

## Supporting information

S1 File Supplementary figures and tables. (PDF)

S2 File Link strengths and classification. (TXT)

S3 File Complete list of suggested RF combinations obtained without any restriction on the TFs considered as candidates. (TXT)
