# Supplementary material for: Exploring induced pluripotency in human fibroblasts via construction, validation, and application of a gene regulatory network
Source: PLoS One. 2019 Aug 2;14(8):e0220742. doi: 10.1371/journal.pone.0220742 (PMC6677386; doi:10.1371/journal.pone.0220742)
Supplement: S1 File — (PDF) [file pone.0220742.s001.pdf]

Exploring induced pluripotency in human fibroblasts via construction, validation, and application of a gene regulatory network

Mehdi Bagheri Hamaneh and Yi-Kuo Yu

Supplentary figures

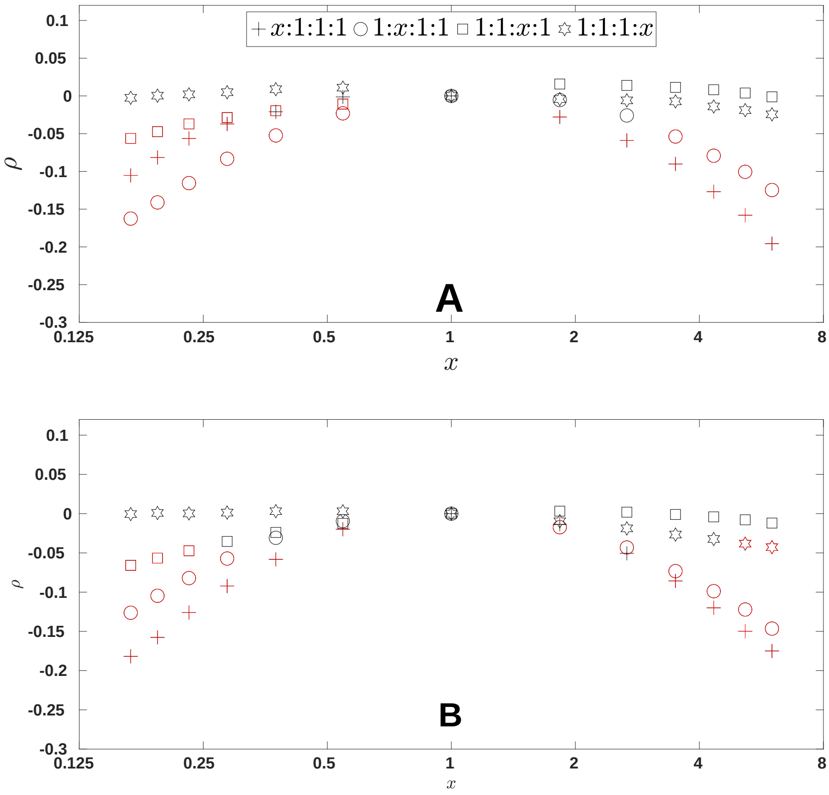

Figure A: The effect of stoichiometry when a network consisting of (A) links in C_2, and (B) links in C_3 is used.

Supplentary tables

Table A: Studies used for training.

series ID	FIB Sample IDs	IPSC Sample IDs	hESC Sample IDs	RFs	Ref.
GSE12390	GSM310854-GSM310856	GSM310838 GSM310839 GSM310844-GSM310850	GSM310860-GSM310862	OSKM	[1]
		GSM310851-GSM310853		OSKMN	
GSE13828	GSM347920	GSM347918	GSM347921-GSM347925	OSNL	[2]
	GSM347919	GSM347916 GSM347917			
GSE14711	GSM366942	GSM367243-GSM367245 GSM367258	GSM367061-GSM367062	OSK	[3]
		GSM367219 GSM367240-GSM367242			
GSE15175	GSM378821	GSM378822-GSM378831	GSM378811-GSM378815	OSKMNL	[4]
GSE16093	GSM402707	GSM402717 GSM402752	GSM402708	OSKM	[5]
		GSM402806			
GSE16654	GSM449728	GSM417794-GSM417796 GSM449730	GSM449729	OSKMN	[6]
GSE9832	GSM248214	GSM248215	GSM248200	OSKM	[7]
	GSM248201 GSM248202	GSM248203			
	GSM248204	GSM248205 GSM248206			
	GSM248209 GSM248210	GSM248211 GSM248212			
GSE9865	GSM249027	GSM249028 GSM249095 GSM249096 GSM249137	GSM249025 GSM249282	OSKMN	[8]
GSE16963	GSM424771	GSM424772 GSM424773		OSK	[9]
GSE26455	GSM649323 GSM649324	GSM649319 GSM649320	GSM649321 GSM649322 GSM649334 GSM649335	OSNL	[10]
	GSM649336 GSM649337	GSM649332 GSM649333			
GSE23583	GSM579884 GSM579885	GSM579903-GSM579906	GSM579898-GSM579902	OSKM	[11]
	GSM579886 GSM579887	GSM579907-GSM579909		OSKML	
	GSM579888 GSM579889	GSM579913-GSM579915			
GSE14897	GSM372142 GSM372144 GSM372146	GSM372157-GSM372159	GSM372150-GSM372152	OSNL	[12]
GSE22167	GSM551186 GSM551187	GSM551192 GSM551193	GSM551200-GSM551203	OSKM	[13]
GSE23968	GSM590393	GSM590394 GSM590395	GSM590391 GSM590392	OSKM	[14]
	GSM590396	GSM590397 GSM590398			
	GSM590399	GSM590400			
	GSM590401 GSM590402	GSM590403 GSM590404			
GSE26672	GSM656447	GSM656442-GSM656446	GSM656448-GSM656451	OSKMNL	[15]
GSE50738	GSM1227447-GSM1227453	GSM1227435-GSM1227442		OSKM	[16]
		GSM1227443-GSM1227446			
GSE22246	GSM553723	GSM553718 GSM553719	GSM553711-GSM553713	OSKM	[17]
		GSM553720 GSM553721			
GSE22392	GSM586150	GSM556996 GSM556997	GSM556994 GSM556995	OSKM	[18]
GSE9709	GSM245341 GSM257524	GSM245339 GSM245342 GSM248216 GSM248217 GSM257520-GSM257523		OSKM	[19]
GSE23402	GSM574058 GSM574059	GSM574080-GSM574085	GSM574061-GSM574077	OSK	[20]

Reprogramming factor abbreviations: O:OCT4, S:SOX2, K:KLF4, M:MYC, N:NANOG, L:LIN28. The dash between two sample IDs indicates that the samples in between the two are also included. For example, GSM310854-GSM310856 means: GSM310854, GSM310855, and GSM310856. Sample GSM248213 from GSE9832 was not included in our analysis because it is reported as being a fibroblast, but it is in fact very similar to an IPSC (the correlation between GSM248213 and the IPSC sample GSM248215 is larger than 0.99).

Table B: Studies used for testing.

**series ID**	**FIB Sample IDs**	**IPSC Sample IDs**	**hESC Sample IDs**	**RFs**	**Ref.**
GSE18226	GSM452726	GSM452727		OSKM	[21]
	GSM452735	GSM452728			
	GSM452730	GSM452729			
	GSM452737	GSM452731			
	GSM452734	GSM452732			
	GSM452736	GSM452733			
GSE24487	GSM603015 GSM603043	GSM603050 GSM603051	GSM603054 GSM603055	OSKM	[22]
	GSM603044 GSM603045	GSM603052 GSM603053			
GSE27924	GSM689384	GSM689066		OSKMNL	[23]
	GSM689395	GSM689366			
	GSM689396	GSM689383			
GSE28815	GSM713542 GSM713543	GSM713544 GSM713545	GSM713549 GSM713587 GSM713554 GSM713590	OSKM	[24]
		GSM713546 GSM713547			
	GSM713560	GSM713575 GSM713577		OSK	
		GSM713581			
GSE37258	GSM914974-GSM914976	GSM914977-GSM914982	GSM914989-GSM914991	OSKM	[25]
	GSM914983-GSM914985	GSM914986-GSM914988			
GSE27206	GSM672187	GSM672181		OSKMNL	[26]
	GSM672188	GSM672182			
	GSM672189	GSM672183			
GSE48830	GSM1185378 GSM1185379	GSM1185380 GSM1185381	GSM1185384 GSM1185385	OSKM	[27]
		GSM1185382 GSM1185383			
GSE55395	GSM1139123 GSM1139124	GSM1139133 GSM1139134		OSKM	[28]
GSE62114	GSM1519865	GSM1519866	GSM1519868	OSKM	[29]
GSE76830	GSM2038536	GSM2038539 GSM2038540	GSM2038538	OSKM	[30]
GSE78716	GSM2073170 GSM2073171	GSM2073174 GSM2073175		OSKM	[31]
	GSM2073172 GSM2073173	GSM2073176 GSM2073177			
GSE62572	GSM1529491	GSM1529489		OSK	[32]
	GSM1529492	GSM1529490			

Reprogramming factor abbreviations: O:OCT4, S:SOX2, K:KLF4, M:MYC, N:NANOG, L:LIN28. The dash between two sample IDs indicates that the samples in between the two are also included. For example, GSM914989-GSM914991 means: GSM914989, GSM914990, and GSM914991.

Table C: **The 4 additional subnetworks.**

Included Classes	L	G^{train}	G^{test}	r^{train}	r^{test}
C_2	633	0.6749	0.6308	0.8106	0.7840
C_3	3393	0.8258	0.7673	0.9028	0.8710
$C_1 \cup C_3$	3838	0.8590	0.7807	0.9224	0.8793
$C_2 \cup C_3$	4026	0.8553	0.7834	0.9203	0.8808

L, G and r denote number of links, goodness of fit, and average correlation respectively.

Table D: **Suggested RF combinations using WN (whole network).**

Rank	RF combination	$\bar{q}$	Rank	RF combination	$\bar{q}$
1	KLF4, MYC, POU5F1, SOX2	0.12184	69	HESX1, MEIS1, POU5F1, SOX2	0.00025
2	KLF4, POU5F1, SOX2	0.08774	70	KLF4, MYC, SOX2, ZIC3	0.00025
3	POU5F1, SOX2	0.05415	71	KLF4, NANOG, ZIC2	0.00023
4	MYC, POU5F1, SOX2	0.03849	72	MEIS1, PRDM14, SOX2	0.00022
5	NANOG, POU5F1, SOX2	0.02922	73	HESX1, SOX2, ZIC3	0.00021
6	KLF4, NANOG, POU5F1, SOX2	0.02629	74	MEIS1, NANOG, POU5F1, SOX2	0.00021
7	MYC, NANOG, POU5F1, SOX2	0.02398	75	HESX1, MYC, POU5F1, ZIC3	0.00021
8	KLF4, POU5F1, PRDM14, SOX2	0.02379	76	EMX2, PRDM14, SOX2, ZIC3	0.00020
9	POU5F1, PRDM14, SOX2	0.02073	77	EMX2, MYC, PRDM14, SOX2	0.00018
10	NANOG, POU5F1, PRDM14, SOX2	0.02070	78	HESX1, NANOG, POU5F1, PRDM14	0.00018
11	MYC, POU5F1, PRDM14, SOX2	0.01822	79	MYC, PRDM14	0.00017
12	KLF4, POU5F1, SOX2, ZIC3	0.01076	80	KLF4, POU5F1, PRDM14	0.00017
13	KLF4, MYC, PRDM14, SOX2	0.00834	81	EMX2, POU5F1, SOX2, ZIC3	0.00017
14	HESX1, POU5F1, PRDM14, SOX2	0.00652	82	KLF4, MEIS1, POU5F1, SOX2	0.00017
15	POU5F1, SOX2, ZIC3	0.00627	83	MYC, POU5F1, ZIC3	0.00016
16	POU5F1, PRDM14, SOX2, ZIC3	0.00585	84	KLF4, POU5F1, PRDM14, ZIC3	0.00015
17	MYC, POU5F1, SOX2, ZIC3	0.00585	85	HESX1, MEIS1, PRDM14, SOX2	0.00014
18	KLF4, MYC, SOX2	0.00574	86	LEF1, PRDM14, SOX2, ZIC3	0.00014
19	HESX1, MYC, POU5F1, SOX2	0.00570	87	MYC, SOX2, ZIC3	0.00013
20	HESX1, POU5F1, SOX2, ZIC3	0.00543	88	MEIS1, MYC, SOX2	0.00012
21	HESX1, KLF4, POU5F1, SOX2	0.00523	89	NANOG, POU5F1, PRDM14, ZIC3	0.00012
22	KLF4, PRDM14, SOX2	0.00510	90	MEIS1, PRDM14, SOX2, ZIC3	0.00012
23	MYC, PRDM14, SOX2	0.00483	91	HESX1, POU5F1, ZIC3	0.00012
24	HESX1, POU5F1, SOX2	0.00428	92	POU5F1, ZIC3	0.00011
25	PRDM14, SOX2	0.00404	93	HESX1, SOX2	0.00011
26	HESX1, NANOG, POU5F1, SOX2	0.00376	94	MYC, NANOG, POU5F1	0.00011
27	POU5F1	0.00281	95	MEIS1, NANOG, PRDM14, SOX2	0.00011
28	MYC, NANOG, PRDM14, SOX2	0.00268	96	HESX1, KLF4, POU5F1, PRDM14	0.00010
29	MYC, POU5F1	0.00259	97	HESX1, MEIS1, MYC, SOX2	0.00010
30	MEIS1, MYC, POU5F1, SOX2	0.00214	98	EMX2, PRDM14, SOX2	0.00010
31	KLF4, NANOG, PRDM14, SOX2	0.00201	99	KLF4, MYC, POU5F1	0.00009
32	HESX1, MYC, PRDM14, SOX2	0.00201	100	MYC, POU5F1, SOX2, ZIC2	0.00009
33	NANOG, PRDM14, SOX2	0.00190	101	HESX1, NANOG, POU5F1, ZIC3	0.00008
34	NANOG, POU5F1, SOX2, ZIC3	0.00181	102	HESX1, MYC, PRDM14, ZIC3	0.00007
35	HESX1, PRDM14, SOX2	0.00147	103	KLF4, MYC, POU5F1, ZIC3	0.00007
36	LEF1, POU5F1, PRDM14, SOX2	0.00140	104	KLF4, MYC, NANOG, SOX2	0.00007
37	MYC, POU5F1, PRDM14	0.00137	105	KLF4, MEIS1, PRDM14, SOX2	0.00007
38	MYC, SOX2	0.00132	106	HESX1, KLF4, POU5F1, ZIC3	0.00006
39	HESX1, KLF4, PRDM14, SOX2	0.00129	107	MYC, PRDM14, SOX2, ZIC2	0.00006
40	MEIS1, POU5F1, PRDM14, SOX2	0.00119	108	EMX2, MYC, POU5F1, SOX2	0.00006
41	HESX1, PRDM14, SOX2, ZIC3	0.00116	109	LEF1, PRDM14, SOX2	0.00006
42	MYC, PRDM14, SOX2, ZIC3	0.00116	110	PRDM14	0.00005
43	KLF4, PRDM14, SOX2, ZIC3	0.00109	111	FOXC1, POU5F1, PRDM14, SOX2	0.00005
44	POU5F1, PRDM14	0.00102	112	KLF4, SOX2, ZIC3	0.00005
45	KLF4, SOX2	0.00101	113	HESX1, MYC, PRDM14	0.00005
46	HESX1, NANOG, PRDM14, SOX2	0.00097	114	KLF4, MEIS1, MYC, SOX2	0.00005
47	PRDM14, SOX2, ZIC3	0.00095	115	EMX2, NANOG, PRDM14, SOX2	0.00005
48	MEIS1, POU5F1, SOX2	0.00091	116	HESX1, MYC, NANOG, SOX2	0.00005
49	MYC, NANOG, POU5F1, PRDM14	0.00081	117	HESX1, MEIS1, SOX2, ZIC3	0.00005
50	KLF4, MYC, POU5F1, PRDM14	0.00061	118	MEIS1, MYC, SOX2, ZIC3	0.00005
51	MYC, POU5F1, PRDM14, ZIC3	0.00055	119	SOX2, ZIC3	0.00005
52	MEIS1, MYC, PRDM14, SOX2	0.00048	120	FOXC1, PRDM14, SOX2, ZIC3	0.00005
53	NANOG, POU5F1, PRDM14	0.00047	121	KLF4, MYC, NANOG, ZIC2	0.00005
54	HESX1, MYC, POU5F1, PRDM14	0.00045	122	HESX1, KLF4, SOX2	0.00005
55	POU5F1, PRDM14, SOX2, ZIC2	0.00042	123	EMX2, MYC, SOX2, ZIC3	0.00005
56	HESX1, POU5F1, PRDM14, ZIC3	0.00042	124	EMX2, HESX1, PRDM14, SOX2	0.00004
57	HESX1, MYC, SOX2, ZIC3	0.00041	125	EMX2, KLF4, PRDM14, SOX2	0.00004
58	SOX2	0.00038	126	HESX1, PRDM14, ZIC3	0.00004
59	HESX1, KLF4, MYC, SOX2	0.00037	127	FOXC1, MYC, PRDM14, SOX2	0.00004
60	EMX2, POU5F1, PRDM14, SOX2	0.00035	128	MEIS1, SOX2	0.00004
61	LEF1, MYC, PRDM14, SOX2	0.00034	129	HESX1, LEF1, PRDM14, SOX2	0.00004
62	MEIS1, POU5F1, SOX2, ZIC3	0.00032	130	HESX1, NANOG, SOX2, ZIC3	0.00004
63	POU5F1, PRDM14, ZIC3	0.00032	131	KLF4, MYC, PRDM14	0.00004
64	HESX1, KLF4, SOX2, ZIC3	0.00032	132	HESX1, MEIS1, SOX2	0.00004
65	HESX1, MYC, SOX2	0.00032	133	HESX1, PRDM14	0.00004
66	NANOG, PRDM14, SOX2, ZIC3	0.00031	134	HESX1, MYC, POU5F1	0.00003
67	NANOG, ZIC2	0.00029	135	KLF4, NANOG, POU5F1, PRDM14	0.00003
68	HESX1, POU5F1, PRDM14	0.00026	136	MYC, PRDM14, ZIC3	0.00001

Table E: **Suggested RF combinations using SUBN2.**

Rank	RF combination	$\bar{q}$	Rank	RF combination	$\bar{q}$
1	KLF4, MYC, POU5F1, SOX2	0.11860	60	HESX1, NANOG, PRDM14, SOX2	0.00026
2	KLF4, POU5F1, SOX2	0.07230	61	KLF4, NANOG, ZIC2	0.00025
3	POU5F1, SOX2	0.05716	62	MYC, NANOG, POU5F1	0.00025
4	MYC, POU5F1, SOX2	0.04038	63	POU5F1, PRDM14, ZIC3	0.00024
5	NANOG, POU5F1, SOX2	0.03588	64	HESX1, POU5F1, PRDM14, ZIC3	0.00022
6	KLF4, POU5F1, PRDM14, SOX2	0.02955	65	KLF4, NANOG, POU5F1, PRDM14	0.00021
7	MYC, NANOG, POU5F1, SOX2	0.02755	66	HESX1, MYC, SOX2	0.00020
8	POU5F1, PRDM14, SOX2	0.02085	67	HESX1, KLF4, MYC, SOX2	0.00020
9	KLF4, NANOG, POU5F1, SOX2	0.01758	68	HESX1, KLF4, POU5F1, PRDM14	0.00019
10	MYC, POU5F1, PRDM14, SOX2	0.01731	69	MEIS1, MYC, POU5F1, SOX2	0.00019
11	NANOG, POU5F1, PRDM14, SOX2	0.01709	70	NANOG, PRDM14, SOX2, ZIC3	0.00018
12	KLF4, MYC, SOX2	0.00612	71	HESX1, MYC, SOX2, ZIC3	0.00018
13	KLF4, MYC, PRDM14, SOX2	0.00601	72	KLF4, MYC, NANOG, ZIC2	0.00018
14	MYC, POU5F1	0.00527	73	HESX1, MYC, POU5F1	0.00017
15	POU5F1, PRDM14, SOX2, ZIC3	0.00484	74	HESX1, MEIS1, POU5F1, SOX2	0.00014
16	MYC, POU5F1, PRDM14	0.00466	75	MYC, SOX2, ZIC3	0.00013
17	POU5F1, PRDM14	0.00429	76	KLF4, MYC, POU5F1	0.00013
18	HESX1, MYC, POU5F1, SOX2	0.00411	77	HESX1, MEIS1, PRDM14, SOX2	0.00012
19	HESX1, POU5F1, PRDM14, SOX2	0.00367	78	KLF4, NANOG	0.00012
20	POU5F1	0.00359	79	MYC, NANOG, ZIC2	0.00012
21	KLF4, MYC, POU5F1, PRDM14	0.00359	80	LEF1, MYC, POU5F1, PRDM14	0.00012
22	KLF4, PRDM14, SOX2	0.00332	81	KLF4, ZIC2	0.00011
23	MYC, POU5F1, SOX2, ZIC3	0.00311	82	ZIC2	0.00011
24	HESX1, POU5F1, SOX2	0.00308	83	MEIS1, PRDM14, SOX2	0.00011
25	MYC, PRDM14, SOX2	0.00299	84	FOXC1, POU5F1, PRDM14, SOX2	0.00010
26	MYC, NANOG, POU5F1, PRDM14	0.00277	85	LEF1, MYC, PRDM14, SOX2	0.00010
27	HESX1, KLF4, POU5F1, SOX2	0.00269	86	KLF4, LEF1, NANOG, ZIC2	0.00010
28	NANOG, POU5F1, PRDM14	0.00255	87	HESX1, SOX2, ZIC3	0.00010
29	PRDM14, SOX2	0.00253	88	MYC, NANOG, SOX2	0.00009
30	POU5F1, SOX2, ZIC3	0.00242	89	KLF4, MYC, SOX2, ZIC3	0.00009
31	LEF1, POU5F1, PRDM14, SOX2	0.00196	90	KLF4, MYC, NANOG, SOX2	0.00006
32	HESX1, NANOG, POU5F1, SOX2	0.00195	91	HESX1, MYC, PRDM14	0.00006
33	MYC, SOX2	0.00182	92	LEF1, NANOG, ZIC2	0.00006
34	MEIS1, POU5F1, PRDM14, SOX2	0.00159	93	HESX1, SOX2	0.00005
35	HESX1, POU5F1, SOX2, ZIC3	0.00148	94	MYC, NANOG, PRDM14	0.00005
36	MYC, NANOG, PRDM14, SOX2	0.00146	95	LEF1, MYC, NANOG, ZIC2	0.00005
37	KLF4, POU5F1, PRDM14	0.00142	96	KLF4, MEIS1, NANOG, ZIC2	0.00005
38	MYC, PRDM14, SOX2, ZIC3	0.00106	97	HESX1, MYC, NANOG, POU5F1	0.00005
39	NANOG, PRDM14, SOX2	0.00102	98	MEIS1, NANOG, ZIC2	0.00005
40	KLF4, NANOG, PRDM14, SOX2	0.00101	99	KLF4, POU5F1, PRDM14, ZIC3	0.00005
41	KLF4, POU5F1, SOX2, ZIC3	0.00094	100	FOXC1, MYC, PRDM14, SOX2	0.00005
42	HESX1, MYC, PRDM14, SOX2	0.00084	101	HESX1, KLF4, MYC, POU5F1	0.00005
43	PRDM14, SOX2, ZIC3	0.00078	102	FOXC1, PRDM14, SOX2, ZIC3	0.00005
44	HESX1, MYC, POU5F1, PRDM14	0.00078	103	HESX1, KLF4, MYC, PRDM14	0.00005
45	KLF4, PRDM14, SOX2, ZIC3	0.00063	104	KLF4, MYC, ZIC2	0.00005
46	HESX1, PRDM14, SOX2	0.00061	105	NANOG	0.00004
47	SOX2	0.00059	106	MEIS1, MYC, POU5F1, PRDM14	0.00004
48	MYC, PRDM14	0.00055	107	HESX1, KLF4, NANOG, ZIC2	0.00004
49	NANOG, POU5F1, SOX2, ZIC3	0.00053	108	HESX1, PRDM14	0.00004
50	HESX1, POU5F1, PRDM14	0.00048	109	HESX1, MYC, NANOG, SOX2	0.00004
51	HESX1, PRDM14, SOX2, ZIC3	0.00047	110	HESX1, POU5F1	0.00004
52	HESX1, KLF4, PRDM14, SOX2	0.00046	111	HESX1, MYC, PRDM14, ZIC3	0.00004
53	MYC, POU5F1, PRDM14, ZIC3	0.00036	112	KLF4, LEF1, ZIC2	0.00004
54	MEIS1, MYC, PRDM14, SOX2	0.00034	113	LEF1, MEIS1, NANOG, ZIC2	0.00004
55	HESX1, NANOG, POU5F1, PRDM14	0.00033	114	SOX2, ZIC3	0.00004
56	NANOG, ZIC2	0.00031	115	LEF1, ZIC2	0.00004
57	PRDM14	0.00031	116	NANOG, POU5F1, PRDM14, ZIC3	0.00004
58	KLF4, MYC, PRDM14	0.00028	117	HESX1, MYC, POU5F1, ZIC3	0.00002
59	KLF4, SOX2	0.00027	118		

Table F: **Suggested RF combinations using SUBN1.**

Rank	RF combination	$\bar{q}$	Rank	RF combination	$\bar{q}$
1	KLF4, MYC, POU5F1, SOX2	0.08187	66	MYC, POU5F1, PRDM14, ZIC3	0.00024
2	KLF4, POU5F1, SOX2	0.07010	67	POU5F1, PRDM14, ZIC3	0.00023
3	POU5F1, SOX2	0.06098	68	MYC, POU5F1	0.00023
4	MYC, POU5F1, SOX2	0.05538	69	KLF4, POU5F1, PRDM14, ZIC3	0.00023
5	MYC, NANOG, POU5F1, SOX2	0.04989	70	MYC, POU5F1, PRDM14	0.00022
6	NANOG, POU5F1, SOX2	0.04710	71	MEIS1, POU5F1, SOX2, ZIC3	0.00022
7	KLF4, NANOG, POU5F1, SOX2	0.04489	72	POU5F1, PRDM14	0.00021
8	NANOG, POU5F1, PRDM14, SOX2	0.01431	73	KLF4, MYC, POU5F1, PRDM14	0.00021
9	KLF4, POU5F1, PRDM14, SOX2	0.01268	74	MYC, NANOG, POU5F1	0.00021
10	HESX1, MYC, POU5F1, SOX2	0.01239	75	HESX1, NANOG, PRDM14	0.00020
11	HESX1, KLF4, POU5F1, SOX2	0.01013	76	MYC, NANOG, POU5F1, ZIC3	0.00020
12	POU5F1, PRDM14, SOX2	0.01001	77	LEF1, POU5F1, PRDM14, SOX2	0.00019
13	HESX1, POU5F1, SOX2	0.00916	78	HESX1, KLF4, NANOG, PRDM14	0.00019
14	HESX1, NANOG, POU5F1, SOX2	0.00904	79	HESX1, KLF4, MYC, PRDM14	0.00018
15	MYC, POU5F1, PRDM14, SOX2	0.00889	80	HESX1, MYC, PRDM14	0.00018
16	HESX1, POU5F1, PRDM14, SOX2	0.00844	81	POU5F1, ZIC3	0.00018
17	KLF4, POU5F1, SOX2, ZIC3	0.00722	82	KLF4, POU5F1, PRDM14	0.00018
18	MYC, POU5F1, SOX2, ZIC3	0.00560	83	HESX1, MYC, PRDM14, ZIC3	0.00017
19	NANOG, POU5F1, SOX2, ZIC3	0.00545	84	MEIS1, MYC, PRDM14, SOX2	0.00016
20	POU5F1, SOX2, ZIC3	0.00538	85	HESX1, KLF4, POU5F1	0.00016
21	HESX1, POU5F1, SOX2, ZIC3	0.00395	86	HESX1, NANOG, PRDM14, ZIC3	0.00016
22	HESX1, MYC, NANOG, POU5F1	0.00315	87	HESX1, PRDM14	0.00015
23	POU5F1, PRDM14, SOX2, ZIC3	0.00313	88	HESX1, MEIS1, POU5F1, PRDM14	0.00015
24	KLF4, NANOG, PRDM14, SOX2	0.00283	89	HESX1, PRDM14, ZIC3	0.00015
25	KLF4, MYC, PRDM14, SOX2	0.00278	90	POU5F1	0.00014
26	MYC, NANOG, PRDM14, SOX2	0.00251	91	MYC, SOX2, ZIC3	0.00014
27	KLF4, PRDM14, SOX2	0.00242	92	NANOG, POU5F1, ZIC3	0.00014
28	MYC, PRDM14, SOX2	0.00236	93	FOXC1, POU5F1, PRDM14, SOX2	0.00013
29	NANOG, PRDM14, SOX2	0.00231	94	KLF4, MYC, POU5F1, ZIC3	0.00013
30	PRDM14, SOX2	0.00210	95	NANOG, POU5F1	0.00012
31	HESX1, NANOG, POU5F1	0.00192	96	MEIS1, PRDM14, SOX2	0.00011
32	HESX1, MYC, POU5F1	0.00156	97	KLF4, MEIS1, PRDM14, SOX2	0.00011
33	HESX1, NANOG, POU5F1, PRDM14	0.00146	98	HESX1, MEIS1, PRDM14, SOX2	0.00011
34	MEIS1, POU5F1, PRDM14, SOX2	0.00133	99	KLF4, POU5F1, ZIC3	0.00010
35	HESX1, MYC, PRDM14, SOX2	0.00124	100	HESX1, KLF4, PRDM14, ZIC3	0.00010
36	HESX1, NANOG, POU5F1, ZIC3	0.00116	101	KLF4, NANOG, POU5F1, ZIC3	0.00010
37	HESX1, NANOG, PRDM14, SOX2	0.00107	102	HESX1, KLF4, PRDM14	0.00010
38	HESX1, KLF4, PRDM14, SOX2	0.00104	103	MEIS1, NANOG, PRDM14, SOX2	0.00010
39	MEIS1, MYC, POU5F1, SOX2	0.00097	104	KLF4, MYC, SOX2, ZIC3	0.00009
40	HESX1, MYC, POU5F1, ZIC3	0.00095	105	EMX2, POU5F1, PRDM14, SOX2	0.00009
41	HESX1, PRDM14, SOX2	0.00094	106	KLF4, MYC, POU5F1	0.00009
42	HESX1, POU5F1	0.00090	107	MEIS1, MYC, POU5F1, PRDM14	0.00006
43	HESX1, MYC, POU5F1, PRDM14	0.00090	108	POU5F1, PRDM14, SOX2, ZIC2	0.00006
44	HESX1, KLF4, MYC, POU5F1	0.00082	109	MYC, SOX2	0.00006
45	HESX1, POU5F1, PRDM14	0.00080	110	HESX1, MYC, SOX2, ZIC3	0.00005
46	HESX1, POU5F1, ZIC3	0.00077	111	FOXC1, POU5F1, SOX2, ZIC3	0.00005
47	HESX1, KLF4, POU5F1, ZIC3	0.00076	112	MEIS1, PRDM14, SOX2, ZIC3	0.00005
48	MEIS1, POU5F1, SOX2	0.00073	113	KLF4, NANOG, ZIC2	0.00004
49	HESX1, KLF4, POU5F1, PRDM14	0.00072	114	SOX2, ZIC3	0.00004
50	KLF4, MEIS1, POU5F1, SOX2	0.00070	115	FOXC1, PRDM14, SOX2, ZIC3	0.00004
51	HESX1, POU5F1, PRDM14, ZIC3	0.00068	116	EMX2, MYC, POU5F1, SOX2	0.00004
52	HESX1, KLF4, NANOG, POU5F1	0.00066	117	EMX2, POU5F1, SOX2, ZIC3	0.00004
53	MYC, PRDM14, SOX2, ZIC3	0.00065	118	EMX2, HESX1, POU5F1, SOX2	0.00004
54	HESX1, PRDM14, SOX2, ZIC3	0.00057	119	MYC, NANOG, PRDM14	0.00004
55	KLF4, PRDM14, SOX2, ZIC3	0.00056	120	HESX1, SOX2, ZIC3	0.00004
56	PRDM14, SOX2, ZIC3	0.00053	121	EMX2, NANOG, POU5F1, SOX2	0.00004
57	NANOG, PRDM14, SOX2, ZIC3	0.00050	122	KLF4, MYC, SOX2	0.00004
58	MYC, NANOG, POU5F1, PRDM14	0.00041	123	MYC, PRDM14	0.00004
59	NANOG, POU5F1, PRDM14	0.00037	124	MYC, NANOG, SOX2, ZIC3	0.00004
60	MEIS1, NANOG, POU5F1, SOX2	0.00034	125	KLF4, MYC, NANOG, POU5F1	0.00004
61	HESX1, MEIS1, POU5F1, SOX2	0.00034	126	MEIS1, NANOG, POU5F1, PRDM14	0.00002
62	KLF4, NANOG, POU5F1, PRDM14	0.00029	127	KLF4, MEIS1, POU5F1, PRDM14	0.00001
63	NANOG, POU5F1, PRDM14, ZIC3	0.00028	128	MEIS1, POU5F1, PRDM14	0.00001
64	HESX1, MYC, NANOG, PRDM14	0.00027	129	KLF4, MYC, NANOG, PRDM14	0.00001
65	MYC, POU5F1, ZIC3	0.00024	130		

Table G: Comparison between average fitted networks obtained using different c_0 values.

c_0	2	4	6	8	10	11	12	13	14	18
F^a	0.1667	0.1476	0.1381	0.1309	0.1312	0.1298	0.1288	0.1291	0.1291	0.1297
r	0.6142	0.7958	0.9013	0.9637	0.9898	0.9977	1.0000	0.9980	0.9957	0.9300
r_{rank}	0.5571	0.7268	0.8526	0.9357	0.9820	0.9946	1.0000	0.9968	0.9925	0.9175

r (r_{rank}) denotes the (rank) correlation between link strengths of the average fitted networks obtained using c_0 and those obtained using $c_0 = 12$.

Table H: Comparison between Gs in the absence and presence of the constraint on the initial values.

Index	1	2	3	4	5	6	7	8	9	10
G (with constraint)	0.8710	0.8711	0.8711	0.8709	0.8711	0.8710	0.8710	0.8713	0.8711	0.8712
G (no constraint)	0.8612	0.8139	0.8496	0.8642	0.7859	0.8577	0.8542	0.8689	0.8458	0.8390

References

[1] Maherali N, Ahfeldt T, Rigamonti A, Utikal J, Cowan C, Hochedlinger K. A high-efficiency system for the generation and study of human induced pluripotent stem cells. Cell Stem Cell. 2008;3(3):340–345.

[2] Ebert AD, Yu J, Rose FF, Mattis VB, Lorson CL, Thomson JA, et al. Induced pluripotent stem cells from a spinal muscular atrophy patient. Nature. 2009;457(7227):277–280.

[3] Soldner F, Hockemeyer D, Beard C, Gao Q, Bell GW, Cook EG, et al. Parkinson's disease patient-derived induced pluripotent stem cells free of viral reprogramming factors. Cell. 2009;136(5):964–977.

[4] Yu J, Hu K, Smuga-Otto K, Tian S, Stewart R, Slukvin II, et al. Human induced pluripotent stem cells free of vector and transgene sequences. Science. 2009;324(5928):797–801.

[5] Kim D, Kim CH, Moon JI, Chung YG, Chang MY, Han BS, et al. Generation of human induced pluripotent stem cells by direct delivery of reprogramming proteins. Cell Stem Cell. 2009;4(6):472–476.

[6] Chin MH, Mason MJ, Xie W, Volinia S, Singer M, Peterson C, et al. Induced pluripotent stem cells and embryonic stem cells are distinguished by gene expression signatures. Cell Stem Cell. 2009;5(1):111–123.

[7] Park IH, Zhao R, West JA, Yabuuchi A, Huo H, Ince TA, et al. Reprogramming of human somatic cells to pluripotency with defined factors. Nature. 2008;451(7175):141–146.

[8] Lowry WE, Richter L, Yachechko R, Pyle AD, Tchieu J, Sridharan R, et al. Generation of human induced pluripotent stem cells from dermal fibroblasts. Proc Natl Acad Sci USA. 2008;105(8):2883–2888.

[9] Oda Y, Yoshimura Y, Ohnishi H, Tadokoro M, Katsube Y, Sasao M, et al. Induction of pluripotent stem cells from human third molar mesenchymal stromal cells. J Biol Chem. 2010;285(38):29270–29278.

[10] Munoz J, Low TY, Kok YJ, Chin A, Frese CK, Ding V, et al. The quantitative proteomes of human-induced pluripotent stem cells and embryonic stem cells. Mol Syst Biol. 2011;7:550.

[11] Warren L, Manos PD, Ahfeldt T, Loh YH, Li H, Lau F, et al. Highly efficient reprogramming to pluripotency and directed differentiation of human cells with synthetic modified mRNA. Cell Stem Cell. 2010;7(5):618–630.

[12] Si-Tayeb K, Noto FK, Nagaoka M, Li J, Battle MA, Duris C, et al. Highly efficient generation of human hepatocyte-like cells from induced pluripotent stem cells. Hepatology. 2010;51(1):297–305.

[13] Loh YH, Hartung O, Li H, Guo C, Sahalie JM, Manos PD, et al. Reprogramming of T cells from human peripheral blood. Cell Stem Cell. 2010;7(1):15–19.

[14] Loewer S, Cabili MN, Guttman M, Loh YH, Thomas K, Park IH, et al. Large intergenic non-coding RNA-RoR modulates reprogramming of human induced pluripotent stem cells. Nat Genet. 2010;42(12):1113–1117.

[15] Hu K, Yu J, Suknuntha K, Tian S, Montgomery K, Choi KD, et al. Efficient generation of transgene-free induced pluripotent stem cells from normal and neoplastic bone marrow and cord blood mononuclear cells. Blood. 2011;117(14):e109–119.

[16] Kamao H, Mandai M, Okamoto S, Sakai N, Suga A, Sugita S, et al. Characterization of human induced pluripotent stem cell-derived retinal pigment epithelium cell sheets aiming for clinical application. Stem Cell Reports. 2014;2(2):205–218.

[17] Tchieu J, Kuoy E, Chin MH, Trinh H, Patterson M, Sherman SP, et al. Female human iPSCs retain an inactive X chromosome. Cell Stem Cell. 2010;7(3):329–342.

[18] Chin MH, Pellegrini M, Plath K, Lowry WE. Molecular analyses of human induced pluripotent stem cells and embryonic stem cells. Cell Stem Cell. 2010;7(2):263–269.

[19] Masaki H, Ishikawa T, Takahashi S, Okumura M, Sakai N, Haga M, et al. Heterogeneity of pluripotent marker gene expression in colonies generated in human iPS cell induction culture. Stem Cell Res. 2007;1(2):105–115.

[20] Guenther MG, Frampton GM, Soldner F, Hockemeyer D, Mitalipova M, Jaenisch R, et al. Chromatin structure and gene expression programs of human embryonic and induced pluripotent stem cells. Cell Stem Cell. 2010;7(2):249–257.

[21] Doi A, Park IH, Wen B, Murakami P, Aryee MJ, Irizarry R, et al. Differential methylation of tissue- and cancer-specific CpG island shores distinguishes human induced pluripotent stem cells, embryonic stem cells and fibroblasts. Nat Genet. 2009;41(12):1350–1353.

[22] Liu GH, Barkho BZ, Ruiz S, Diep D, Qu J, Yang SL, et al. Recapitulation of premature ageing with iPSCs from Hutchinson-Gilford progeria syndrome. Nature. 2011;472(7342):221–225.

[23] Lapasset L, Milhavet O, Prieur A, Besnard E, Babled A, Ait-Hamou N, et al. Rejuvenating senescent and centenarian human cells by reprogramming through the pluripotent state. Genes Dev. 2011;25(21):2248–2253.

[24] Valamehr B, Abujarour R, Robinson M, Le T, Robbins D, Shoemaker D, et al. A novel platform to enable the high-throughput derivation and characterization of feeder-free human iPSCs. Sci Rep. 2012;2:213.

[25] Ma Y, Li C, Gu J, Tang F, Li C, Li P, et al. Aberrant gene expression profiles in pluripotent stem cells induced from fibroblasts of a Klinefelter syndrome patient. J Biol Chem. 2012;287(46):38970–38979.

[26] Corti S, Nizzardo M, Simone C, Falcone M, Nardini M, Ronchi D, et al. Genetic correction of human induced pluripotent stem cells from patients with spinal muscular atrophy. Sci Transl Med. 2012;4(165):165ra162.

[27] Awe JP, Lee PC, Ramathal C, Vega-Crespo A, Durruthy-Durruthy J, Cooper A, et al. Generation and characterization of transgene-free human induced pluripotent stem cells and conversion to putative clinical-grade status. Stem Cell Res Ther. 2013;4(4):87.

[28] Lu J, Li H, Hu M, Sasaki T, Baccei A, Gilbert DM, et al. The distribution of genomic variations in human iPSCs is related to replication-timing reorganization during reprogramming. Cell Rep. 2014;7(1):70–78.

[29] Shimamoto A, Kagawa H, Zensho K, Sera Y, Kazuki Y, Osaki M, et al. Reprogramming suppresses premature senescence phenotypes of Werner syndrome cells and maintains chromosomal stability over long-term culture. PLoS ONE. 2014;9(11):e112900.

[30] Matsumoto T, Fujimori K, Andoh-Noda T, Ando T, Kuzumaki N, Toyoshima M, et al. Functional Neurons Generated from T Cell-Derived Induced Pluripotent Stem Cells for Neurological Disease Modeling. Stem Cell Reports. 2016;6(3):422–435.

[31] Lu J, Li H, Baccei A, Sasaki T, Gilbert DM, Lerou PH. Influence of ATM-Mediated DNA Damage Response on Genomic Variation in Human Induced Pluripotent Stem Cells. Stem Cells Dev. 2016;25(9):740–747.

[32] Fujie Y, Fusaki N, Katayama T, Hamasaki M, Soejima Y, Soga M, et al. New type of Sendai virus vector provides transgene-free iPS cells derived from chimpanzee blood. PLoS ONE. 2014;9(12):e113052.
